# Application of electrostatic and multi-layer embedding in Hirshfeld atom refinements: a cost-effective approach for approximating bulk effects in crystalline environments

**DOI:** 10.1107/S160057672600717X

**Published:** 2026-09-04

**Authors:** Bruno Landeros-Rivera, Florian Kleemiss

**Affiliations:** a Departamento de Química Inorgánica, Facultad de Química, Universidad Nacional Autónoma de México, Mexico; b Institute of Inorganic Chemistry, RWTH Aachen University, Landoltweg 1a, 52074 Aachen, Germany; Shiv Nadar Institution of Eminence, India

**Keywords:** *NoSpherA2*, Hirshfeld atom refinement, quantum mechanics/molecular mechanics, electrostatic embedding

## Abstract

One of the main advantages of Hirshfeld atom refinement (HAR), besides improved refinement statistics and reduced uncertainties of all derived parameters, is the possibility of refining anisotropic hydrogen atoms without any type of restriction to a correct distance. Notwithstanding this, there are still some problems that need attention for an accurate refinement of H atoms. For instance, *X*—H bond distances (*X* = N, O) are typically underestimated when they participate in a strong hydrogen bond and the crystallographic environment is not accounted for in the refinement model. In this work, we propose the use of an embedding scheme to model bulk effects approximately, where a molecule or group of molecules is polarized by electrostatic potential (ESP)-derived charges. Quantum mechanics/molecular mechanics methods based on ESP-derived charges have been used successfully to reproduce band gaps or optical and electronic properties of crystalline systems. It is demonstrated that the H-atom refinement parameters obtained with this approach are comparable to those obtained from pure quantum mechanical models, but with a significantly lower computational cost. Thus, this embedded HAR method is suitable for performing refinements in reasonable time frames for large systems in which strong intermolecular interactions, such as hydrogen bonds, exist.

## Abstract 2

A new efficient embedding mechanism for molecule-in-a-crystal electron-density calculations during Hirshfeld atom refinement is made available in *NoSpherA2* and benchmarked.

## 1. Introduction

Hirshfeld atom refinement (HAR) (Jayatilaka & Dittrich, 2008 ▸; Capelli *et al.*, 2014 ▸) has become an essential tool for modelling non-spherical atomic densities in crystallographic refinements, allowing accurate structure determination from diffraction experiments. One of the advantages of HAR is the possibility of refining hydrogen atoms anisotropically without using any type of constraints or restraints, or relying on databases, provided that sufficiently good quality data are collected (Woińska *et al.*, 2014 ▸; Woińska *et al.*, 2016 ▸; Woińska *et al.*, 2021 ▸; Xu *et al.*, 2023 ▸). This indicates that the problem of refining hydrogen atoms is not only the quality of the diffraction experiment itself (the relatively weak signal of this type of atom) but also the approximations used, for example the independent atom model (IAM), or the correlations occurring when refining the electron-density model simultaneously. The systematic IAM-associated bond-length errors (determined with respect to neutron diffraction reference values) can be one order of magnitude larger for H atoms (Fischer *et al.*, 2021 ▸). Thus, HAR provides a much more realistic model for *X*—H bonds than the IAM.

Determining the precise positions of hydrogen atoms in a crystal structure is essential to understanding the role of hydrogen bonds in the assembly of supramolecular adducts (Thompson & White, 2023 ▸), molecular recognition in bio­logical systems (Bulusu & Desiraju, 2020 ▸), the thermal and rheological properties of minerals (Gatta *et al.*, 2021 ▸), the hydrogen storage capacity of materials (Woińska *et al.*, 2023 ▸), or conductivity in superprotonic crystals (Makarova *et al.*, 2021 ▸).

It has been shown that it is not sufficient to consider only intramolecular covalent *X*—H bonding during the calculation of scattering factors when the H atom is also involved in other types of non-covalent interaction, such as hydrogen bonds or agostic interactions (Dittrich *et al.*, 2012 ▸; Wieduwilt *et al.*, 2020*a* ▸; Ruth *et al.*, 2022 ▸; Landeros-Rivera *et al.*, 2023 ▸). For appropriate modelling, the crystalline environment must be taken into account, especially when *X* is a highly electronegative atom, such as oxygen or nitrogen. There are many ways this can be achieved. One way is to perform HAR with periodic boundary calculations. Early work in this direction used plane-wave-based or real-space grid methods (Wall, 2016 ▸; Ruth *et al.*, 2022 ▸). While these methods have proven successful, they do not adequately describe the core density, which is predominant in high-resolution X-ray diffraction signals (Davidson *et al.*, 2022 ▸), and this could have an impact on studies where high-resolution data are collected (values lower than 0.5 Å) and where core-polarization or relativistic effects are important (Fischer *et al.*, 2021 ▸; Bučinský *et al.*, 2019 ▸; Pawledzio *et al.*, 2021 ▸; Woińska *et al.*, 2023 ▸). Furthermore, the behaviour of electron density obtained with plane-wave-based programs has been observed to vary, depending not only on the calculation parameters but also on the specific code used (Landeros-Rivera *et al.*, 2025 ▸). Alternatively, a variant of HAR has been developed that uses periodic calculations with Bloch functions of atom-centred Gaussian orbitals, which also shows promising results (Chu *et al.*, 2026 ▸). Despite this, the major drawback of using solid-state programs is their time-consuming nature. These approaches require testing of the convergence of the electron density with respect to several parameters, such as the number of *k*-points, the cutoff energy or the basis set (in plane-wave and localized basis programs, respectively), the fast Fourier transform (FFT) grid, and the selection of the density functional (the use of hybrid functionals significantly increases computation time, becoming practically unfeasible with current methods and personal computers). Therefore, their use is unsuitable for routine refinements where the only objective is to obtain accurate structural parameters.

Another possibility for considering bulk effects is to use a cluster of molecules during the quantum chemical calculation where at least the first coordination sphere is taken into account. This approach is limited to molecular solids, since ionic crystals will require significantly larger clusters because of the presence of long-range interactions. While this approach is affordable for small or medium-sized molecules, it could be prohibitive for large systems, such as coordination complexes, for which the cluster would quickly require more than 500 atoms, along with all-electron relativistic calculations. Therefore, a more efficient and broad approach is necessary for modelling the crystalline environment. Implicit solvent models have also been applied to simulate bulk effects via the conductor-like polarizable continuum model (Brüx *et al.*, 2026 ▸). The hypothesis behind this idea is that, for instance, a hydrogen-bond network in a crystalline system can be compensated by the continuous dielectric medium of a polar solvent such as water. This has been tested for a set of 14 amino acids, showing positive results. Notwithstanding this, a crystal-field model with a more accurate directed representation of the surrounding molecules is desirable.

On this basis, the use of multiscale methods has emerged as an alternative to model bulk effects in HAR. In the first application of this type, which is implemented in *Tonto* (Jayatilaka & Grimwood, 2003 ▸), a quantum chemical calculation of a molecular unit is performed. This is subsequently embedded by a cluster of charges and dipoles that are generated by the Hirshfeld partition of the electron density obtained from the initial calculation (applying symmetry operations), if the user chooses, until convergence of the charges is reached (Jayatilaka & Dittrich, 2008 ▸). This approach has been successfully employed to refine hydrogen-atom positions (Woińska *et al.*, 2016 ▸) and to account properly for the effects of the crystal field (Dittrich *et al.*, 2012 ▸). The same physical principle to model crystal-field effects is used in another methodology implemented in *DiSCaMB* (Chodkiewicz *et al.*, 2018 ▸), which instead of iteratively updating the Hirshfeld charges and dipoles in the self-consistent field (SCF), updates them along with the structure factors after each refinement cycle (Chodkiewicz *et al.*, 2022 ▸) (using the geometry from the previous cycle). Therefore, when used with the appropriate software, this method is expected to be faster, especially if employed with a fragmentation scheme, and it has been efficiently used to refine metal–organic compounds as well as covalent organic frameworks (Woińska *et al.*, 2023 ▸; Woińska *et al.*, 2025 ▸). The use of dipoles in these methods is essential to counteract the shortcomings of Hirshfeld charges (Elking *et al.*, 2012 ▸), which will be discussed later.

Alternatively, another full quantum chemical multi-scale method was developed in which a reference crystal unit, treated at a quantum mechanical (QM) level, is embedded by symmetry-generated units described with extremely localized molecular orbitals (ELMOs) (Genoni, 2013 ▸; Wieduwilt *et al.*, 2020*a* ▸; Macetti *et al.*, 2021 ▸). It was tested for xylitol, providing very accurate O—H refined values when compared with neutron diffraction values. Additionally, a third layer of point charges obtained from the general AMBER force field was tested (Wang *et al.*, 2004 ▸), but it was found that its effect was negligible, so the authors concluded that the single QM/ELMO method is sufficient to model the crystalline field properly. The major drawback of the QM/ELMO refinement technique is that it depends on a database of orbitals (Meyer *et al.*, 2016 ▸; Meyer & Genoni, 2018 ▸) that were pre-computed for certain model systems and basis sets, and thus there may not be suitable ELMOs for all systems. Additionally, since the implementation was made in a modified version of *GAUSSIAN09* (Frisch *et al.*, 2009 ▸), its applicability to the wider community is very restricted for the moment. Therefore, other embedding schemes that can similarly and efficiently describe crystal-field effects in a fast, reliable and widely available framework are desirable.

In this work, we propose the use of an electrostatic embedding scheme, where a molecule or group of molecules is polarized by surrounding electrostatic-potential-derived charges. These are obtained by fitting atom-centred charges to the electrostatic potential surface of the central unit, which is computed with a quantum mechanical method (Chirlian & Francl, 1987 ▸). Particularly, the charges from electrostatic potentials are obtained using a grid-based method (given the acronym CHELPG), which is employed to compute atomic charges fitted to reproduce the electrostatic potential at selected points around the molecule (Breneman & Wiberg, 1990 ▸). In contrast to Hirshfeld charges, CHELPG charges are sufficient to reproduce *ab initio* computed dipoles without the need for higher-order multipoles (Elking *et al.*, 2012 ▸). This embedding scheme has been exhaustively tested for simulating the crystalline environments in molecular and reticular solids (Bjornsson & Bühl, 2012 ▸; Dittmer *et al.*, 2019 ▸), and has successfully been applied to reproduce important bulk properties such as band gaps (Dittmer *et al.*, 2019 ▸), electronic, optical (Shafei *et al.*, 2022 ▸) or vibrational (de Abrantes *et al.*, 2022 ▸) characteristics, and NMR shielding constants (Dittmer *et al.*, 2020 ▸), among others. Therefore, this method is also expected to be applicable for modelling the crystalline environment to obtain improved electron densities in HAR.

This work is divided into three sections. In the first, the new method called CHELPG-embedded HAR (ChE-HAR) is tested for molecular crystals where hydrogen bonds are present. Next, the crystal of an inorganic salt is analysed to determine the capability of ChE-HAR to refine ionic systems. Finally, ChE-HAR is applied to the xylitol crystal structure, comparing the results with those obtained from other embedding schemes and periodic HAR implementations.

## 2. Methodology

### 2.1. The CRYSTAL-QMMM methods

Starting from *ORCA* (Version 5.0; Neese, 2022 ▸), two quantum mechanics/molecular mechanics (QMMM) methods have been implemented to simulate crystalline environments. In the first one, called MOL-CRYSTAL-QMMM (Bjornsson & Bühl, 2012 ▸), the QM region is embedded in a force field that consists of Lennard–Jones potentials that use universal force field (UFF) van der Waals parameters (Rappé *et al.*, 1992 ▸) as well as atomic point charges. In the IONIC-CRYSTAL-QMMM method the QM region is embedded only in a point-charge field. In both methods, atomic CHELPG point charges are computed and updated self-consistently until convergence is reached between the QM and MM zones. However, in its current implementation, the initial atomic point charges of the MOL-CRYSTAL-QMMM method are calculated from Mulliken charges with the GFN2-xtb semiempirical method (Bannwarth *et al.*, 2019 ▸), which requires the presence of the *xtb* executable (Bannwarth *et al.*, 2021 ▸) in the *ORCA* installation folder. In contrast, in the IONIC-CRYSTAL-QMMM method, a single-point calculation with the selected level of theory for the QM region is performed to obtain the initial atomic charges, which generally means it takes longer. A boundary region between QM and MM is introduced by means of electrostatic core potentials to mitigate possible surface effects caused by unsaturated bonds in the QM region, to preserve local orbital symmetries, and to prevent artificial charge transfer between the quantum cluster and point charges. This last is particularly useful for reticular ionic systems. This is automatically handled by *ORCA*.

### 2.2. Implementation of the method in *NoSpherA2*

*NoSpherA2* is a program used to calculate non-spherical form factors by applying the Hirshfeld partition of the electron density obtained from molecular QM calculations (Kleemiss *et al.*, 2021 ▸). It is implemented in *Olex2* (Dolomanov *et al.*, 2009 ▸) and can be interfaced with other quantum mechanical programs, of which *ORCA* (Neese *et al.*, 2020 ▸) has been the one most tested. The input choices in the *NoSpherA2* GUI of *Olex2* are applied using the *Olex2* command-line pack command to grow the system to the selected radius. Two coordinate files are written and used to generate the input for *ORCA*: the QM unit, which is seen when the refinement is started, and the set of atoms surrounding the atoms that make up the MM region. The required call of the MM preparation tool of *ORCA* is performed from within *Olex2*. In the case of MOL-CRYSTAL-QMMM calculations, no additional input is required. In the case of IONIC-CRYSTAL-QMMM, the charge on common ions like alkali, alkali earth and halogen atoms is provided automatically using the -CEL command-line argument during the preparation of input files for *ORCA*.

With the MM input files written automatically, the *ORCA* calculation can proceed normally. A set of default values is placed in the %qmmm block of the input file; the number of QM and effective core potential (ECP) layers (only applicable to IONIC calculations) selected in the GUI, and the force-field filename, are included in the input file. However, no ECPs are defined for hydrogen atoms, so using the IONIC embedding with ECPs is only suitable for structures without hydrogen atoms. The application of the MOL-CRYSTAL-QMMM and IONIC-CRYSTAL-QMMM methods to HAR will be referred to as MChE-HAR and IChE-HAR, respectively.

### 2.3. Application of ChE-HAR to hydrogen-bonded systems

For testing the method, an in-depth analysis was performed on the crystal structure of carbamazepine (Sovago *et al.*, 2016 ▸) for the following reasons: (i) X-ray diffraction data are available up to a resolution of 0.4 Å, (ii) neutron diffraction values are also available, taken at the same temperature, and (iii) it is a relatively large organic molecule (30 atoms) that forms strong N—H⋯O bonds, for which the use of a cluster to model the crystalline environment will require at least 17 molecules to cover the first coordination sphere, making the refinement unfeasible without supercomputing power or GPU-accelerated QM code. It was one of the first examples for which it was reported that taking the crystalline environment into account for a proper refinement of hydrogen atoms is necessary, particularly if these are involved in hydrogen bonding. However, beyond the quantum chemical model and the treatment of the crystalline environment, other factors such as the partitioning scheme and the quality/resolution of the data (Chodkiewicz & Woźniak, 2025 ▸) also affect the condition of the refined parameters of the hydrogen atoms (especially of the anisotropic displacement parameters). In this work, though, the focus is on comparing the results obtained by introducing or omitting approximate bulk effects.

To test the ChE-HAR method further, crystals of urea [CO(NH_2_)_2_; Birkedal *et al.*, 2004 ▸; Swaminathan *et al.*, 1984 ▸], oxalic acid dihydrate [(COOH)_2_·2H_2_O; Kamiński *et al.*, 2014 ▸] and ammonia (NH_3_; Boese *et al.*, 1997 ▸; Hewat & Riekel, 1979 ▸), for which X-ray and neutron diffraction data taken at the same temperature are available, were also examined. For the case of ammonia the experiment corresponds to neutron powder diffraction of ND_3_, so a small difference of about 0.004 Å with respect to NH_3_ in the X-ray data should be expected (Chu *et al.*, 2026 ▸). Different types of hydrogen bond, namely N—H⋯O, O—H⋯O and N—H⋯N, are present in the other systems. We also analysed crystals of salicylic acid (HOC_6_H_4_COOH), sarcosine (CH_3_NHCH_2_COOH), alanine–methionine [CH_3_CH(NH_2_)COOH–CH_3_SCH_2_CH_2_CH(NH_2_)­COOH] and hydrazine borane (N_2_H_4_BH_3_), which show several additional types of hydrogen bonding but do not have neutron reference values available. The X-ray diffraction data were taken from Woińska *et al.* (2016 ▸) and the bond lengths were compared with the suggested neutron diffraction mean values (Allen & Bruno, 2010 ▸), as done in the original study. Lastly, crystals of biphenyl (C_6_H_5_–C_6_H_5_) and diborane (B_2_H_6_) were examined to see the effect of the method on the refinement of structures where nearly nonpolar C—H and B—H bonds are present and where there is no (classical) hydrogen bonding.

### 2.4. Application of ChE-HAR to an ionic system

When analysing salts, some degree of arbitrariness is introduced in the definition of the cluster, since there are at least two different chemical species from which to construct it. For a proper description of the crystalline environment, a large cluster should be defined, which completely covers (at least the first coordination sphere of) all the involved atomic or molecular entities. Cluster construction becomes more difficult when the ions consist of several atoms, as in salts of inorganic coordination complexes, because obtaining a neutral model is non-trivial. Furthermore, electrostatic interactions are long range, requiring even larger clusters than crystals of neutral molecules. Thus, for these cases, the cluster approach in HAR is unfeasible except for tiny molecules. IChE-HAR can therefore be helpful in these situations, as will be exemplified here with the case of magnesium bis(hydrogen maleate) hexahydrate (Mg-HM) whose data, collected at 15 K, were taken from Malaspina *et al.* (2020 ▸).

In Mg-HM, the asymmetric unit is composed of a maleate anion and half an Mg(H_2_O)_6_^2+^ entity (Fig. 7). Since the Mg^2+^ cation lies on an inversion centre, the asymmetric unit is not neutral but has one positive charge. Therefore, a second maleate anion should be employed in the refinement model to ensure neutrality. The first maleate anion forms an O—H⋯O bond with one of the six hydrogen atoms of the three water molecules of the asymmetric unit. Hence, there are five different positions (shown in Fig. 7) where the second maleate anion can be placed. Using a model where all the water molecules of the asymmetric unit are forming hydrogen bonds is not recommended because an excess of negative charges would be introduced, requiring more Mg(H_2_O)_6_^2+^ ions to balance them. For these reasons, we performed five refinements by placing the second maleate anion in each of the remaining five positions. These refinements will be referred to as **I**, **II**, **III**, **IV** or **V**, following the name of each position as defined in Fig. 7. The O—H bonds of the water molecules are compared with those obtained from neutron diffraction data (Malaspina *et al.*, 2017 ▸) collected at a very similar temperature (12 K).

### 2.5. Comparison of ChE-HAR with other methods

The xylitol crystal structure was selected to compare the performance of ChE-HAR with that of the point-charge/dipole model using Hirshfeld partitioning (Dittrich *et al.*, 2012 ▸), referred to here as PCD, and the QM/ELMO multiscale method (Macetti *et al.*, 2021 ▸) described in the *Introduction*[Sec sec1]. ChE-HAR is also tested against three refinement methods that use periodic QM calculations to generate the static electron-density model: (i) a HAR method based on projector augmented wave density functional theory (DFT) calculations (PAW-HAR), implemented in a program called *xHARPy* (Ruth *et al.*, 2022 ▸), (ii) a refinement strategy that uses localized basis set solid-state calculations to generate multipoles that are refined iteratively using the Hansen–Coppens method (Hansen & Coppens, 1978 ▸), implemented in a program called *ReCrystal* (Patzer & Lehmann, 2025 ▸), and (iii) a HAR method based on atom-centred Gaussian orbitals, named pHAR (Chu *et al.*, 2026 ▸), implemented in *Tonto*. Xylitol has already been studied using the methods mentioned above, except for PCD for which the application is performed in this work. The rest of the information will be taken directly from the corresponding publications. We employed the same data as in the previous work (Madsen *et al.*, 2004 ▸), for which X-ray and neutron diffraction results were collected at the same temperature (122 K).

### 2.6. Structure refinement strategies

All the refinements were performed with *Olex2* (Dolomanov *et al.*, 2009 ▸), using *olex2.refine* for the least-squares procedure, *NoSpherA2* for the non-spherical atomic form factors computation (Kleemiss *et al.*, 2021 ▸) and *ORCA* (Version 5.0.4; Neese *et al.*, 2020 ▸; Neese, 2022 ▸) for the wavefunction calculations. The starting parameters were taken from an IAM refinement. The QM approach is indicated by the selected *ab initio* method, Hartree–Fock (HF) or density functional (DF), along with a subscript that specifies the approximation used for modelling the crystalline environment: ‘mol’ stands for a normal HAR with a single calculation unit with no bulk effects introduced, ‘dim’ for the use of a dimer as the calculation unit, ‘clust’ for an explicit cluster of molecules treated with the selected QM method, ‘PCD’ for the point-charge/dipole model using Hirshfeld partitioning with a radius of 8 Å, and ‘MChE’ or ‘IChE’ for the molecular and ionic versions of ChE-HAR, respectively. Representative exam­ples of GGA (BLYP) (Becke, 1988 ▸; Lee *et al.*, 1988 ▸), MetaGGA (r2SCAN) (Furness *et al.*, 2020 ▸), Hybrid (PBE0 and B3LYP) (Adamo & Barone, 1999 ▸; Becke, 1993 ▸), MetaHybrid (M06-2X) (Zhao & Truhlar, 2008 ▸) and range-separated (wB97X) (Chai & Head-Gordon, 2008 ▸) DFs were selected. The def2-TZVP basis was employed, which is recommended for polar organic molecules (Brüx *et al.*, 2026 ▸), except for the refinements of the xylitol structure, where the cc-pVTZ basis set was employed to make a more appropriate comparison with the QM/ELMO results that were obtained with this basis function. All atoms were refined anisotropically without any type of constraint or restraint. The refinements were carried out on a laptop with a Ryzen 5 3500 processor, using six CPUs and 10 GB of RAM (these criteria can be selected directly in the *NoSpherA2* GUI in *Olex2*).

To investigate quantitatively the effect of the different refinement strategies on the anisotropic displacement parameters (ADPs), the similarity index *S*_12_ (Whitten & Spackman, 2006 ▸) of the ADPs for all refinements against the neutron diffraction reference was calculated. *S*_12_ is a metric that measures the overlap between two ADP tensors that depends on the magnitude of the mean-square displacements and the orientations of the principal axes of the displacement ellipsoid. A value of *S*_12_ = 0 indicates perfect agreement, and it increases proportionally with the discrepancy between the ADPs.

## 3. Results

### 3.1. Carbamazepine

#### 3.1.1. Refinement of *X*—H bonds with different models of crystal environment

In this section, the application of the molecular and ionic embedding QMMM schemes to HAR (MChE-HAR and IChE-HAR, respectively) is analysed. Particular attention is paid to the refined N1—H1*B* bond length (Fig. 1 ▸), which is most affected by the crystalline environment because of the formation of the strong N—H⋯O hydrogen bond. The refined N1—H1*B* bond lengths using the B3LYP functional with different refinement strategies are shown in Fig. 2 ▸. Standard uncertainties (s.u.s) are also shown as error bars. The largest deviation with respect to the neutron diffraction value is obtained with B3LYP_mol_. Using an explicit cluster does not produce a closer agreement with the neutron diffraction data than using a dimer as defined in Fig. 1 ▸, since B3LYP_dim_ and B3LYP_clust_ converge to the same values, although the employed cluster (six surrounding molecules) was not big enough to complete the first coordination sphere. It was not possible to use larger clusters because they require more memory than the computer equipment used in this work can accommodate.

For carbamazepine, MChE-HAR and IChE-HAR yield results that, compared with neutron diffraction data, are statistically indistinguishable from those of the cluster model within 1 s.u. Moreover, there is no noteworthy difference in the *R*_1_ residuals of each HAR procedure (all values oscillate between 0.0393 and 0.0395). B3LYP_IChE_ and B3LYP_MChE_ were repeated using radii of 18 and 24 Å, but no differences were observed, indicating that 12 Å seems satisfactory for modelling bulk effects in small neutral organic molecules. The deviation between the refined N1—H1*B* bond lengths and the neutron diffraction value for B3LYP_dim_, B3LYP_clust_, B3LYP_MChE_ and B3LYP_IChE_ is (on average) 0.033 Å. For the N1—H1*A* and C—H bond lengths, the mean absolute deviations with respect to the neutron diffraction values are smaller (about 0.010 Å, Table S1), which is expected for low-polarity bonds (Chu *et al.*, 2026 ▸).

#### 3.1.2. Refinement of ADPs with different models of crystal environment

In relation to thermal motion, *S*_12_ values of the ADPs for all refinements against the neutron diffraction reference were calculated. The average across all atoms and the individual contributions of hydrogen and non-hydrogen atoms are shown in Fig. 3 ▸. The full table of differences and peanut plots are shown in Table S5 and Fig. S4, respectively, in the supporting information. Just as with the N1—H1*B* bond length, all the HAR procedures where bulk effects were introduced reach similar *S*_12_ values in all cases. B3LYP_mol_ shows a greater deviation from the neutron diffraction ADPs. However, the comparison of *S*_12_ obtained for atom H1*B* with B3LYP_mol_ (3.53), B3LYP_dim_ (0.75), B3LYP_clust_ (1.19), B3LYP_MChE_ (1.92) and B3LYP_IChE_ (1.58) reveals that, although ChE-HAR methods show an improvement over the case where the crystal environment is ignored, the ADPs obtained with this method­ology are less similar to the neutron ADPs than those obtained with purely quantum bulk models.

#### 3.1.3. IChE-HAR of *X*—H bonds and ADPs with different density functionals

The influence of the QM approach on the N1—H1*B* bond length of carbamazepine was assessed. The results are shown in Fig. 4 ▸. Although the difference with respect to the neutron diffraction value is slightly smaller for BLYP, all the DF methods reach the same result within 1 s.u. The only method that reached a value comparable to that from neutron diffraction (within 1 s.u.) is Hartree–Fock.

The similarity index between the neutron reference and the ADPs obtained after refinement using different DF methods is presented in Fig. 5 ▸. The data for all atoms are presented in Table S7 in the supporting information. The worst agreement is obtained with HF, while the rest of the DF methods behave very similarly. This emphasizes that the deconvolution of the atomic density from the displacement parameters is crucial when feedback from experimental data is to be incorporated into, for example, fitted wavefunctions (Genoni & Sironi, 2025 ▸) so as not to introduce bias from improper modelling of the displacement factors into the static density and hence the resulting fitted wavefunction.

#### 3.1.4. Comparison of refinement times

One important aspect to examine is the total time (in hours) that the QM calculations took for each refinement method (the time taken by *NoSpherA2* and *olex2.refine* is negligible in comparison). These are shown in Fig. 6 ▸. It is evident that the ChE-HAR methods show the best cost/benefit relationship to model the crystalline environment, since they reproduce the same results as the explicit cluster approach but reduce the computing time by 90%. Although B3LYP_dim_ took about half an hour less than the ChE-HAR methods, this approach will not be useful for other systems in general where several hydrogen atoms might be involved in strong interactions. In the original work (Sovago *et al.*, 2016 ▸) a cluster of charges and dipoles in the HAR method was applied to refine carbamazepine with BLYP and the cc-pVTZ basis set, which took 27 h on a 16-core server. Although the comparison cannot be straightforward because there is no available information about the processor or the amount of memory employed in that refinement, it is notable that IChE-HAR, performed with the same DF and basis set, reached a closer agreement with the neutron diffraction value (with a difference of 1 s.u.) but took only 38.4 min with six cores. We repeated the refinement with *DiSCaMB*-HAR, applying the same level of theory and a cut-off radius of 12 Å for the Hirshfeld charges and dipoles (using *ORCA* for the calculation of the QM unit) on the same equipment. We obtained the same results as reported by Sovago *et al.* (2016 ▸) but it took only 8.3 min, confirming that avoiding iteratively updating the Hirshfeld charges and dipoles with the SCF can speed up calculations (Fig. S5). As expected, IChE-HAR with more elaborate DFs, especially hybrid functionals such as M06-2X and wB97X, took a longer time without any benefit.

### 3.2. Other hydrogen-bonded systems

The application of IChE-HAR to the other selected crystals confirms some of the findings for carbamazepine. For the non-polar biphenyl and diborane systems, B3LYP_mol_ and B3LYP_IChE_ lead to essentially the same *X*—H bond lengths (difference smaller than 1 s.u.), except for one C—H bond in biphenyl, where the hydrogen atom is involved in inter- and intramolecular H⋯H interactions. The same trend was found for the rest of the *X*—H bonds in the crystal structures of the other compounds that do not participate in strong intermolecular interactions. In oxalic acid dihydrate and salicylic acid, there are O—H⋯O hydrogen bonds that are already taken into account in the QM region, so the electrostatic embedding has no significant effect either. On the other hand, in those cases where the hydrogen atoms form strong O—H⋯O, N—H⋯O or N—H⋯O hydrogen bonds, with donor–H–acceptor angles larger than 130° (Desiraju & Steiner, 2001 ▸), a significant change in the *X*—H bond length is observed (at least more than 1 s.u.) when the crystalline field is introduced. The mean absolute deviations (MADs) with respect to the neutron diffraction values are shown in Fig. S1. It is observed that in all cases the MAD with IChE-HAR is reduced at least by half, although the reduction can be more significant, exemplified by the two analysed amino acids (sarcosine and alanine–methionine).

### 3.3. Salts/coordination complexes

#### 3.3.1. IChE-HAR of water O—H bond lengths and ADPs

For the water molecule forming the hydrogen bond within the asymmetric unit of Mg-HM, the absolute deviation with respect to the neutron diffraction value is of the order of 10^−3^ Å (Table S3). However, no systematic improvement was observed for the rest of the O—H bonds. For instance, when the maleate anion is placed in position **II**, the absolute deviation for this bond is larger than when it is placed elsewhere. Alternatively, the effect of introducing the crystal field through IChE-HAR was analysed (**I**_IChE_ to **V**_IChE_). Similarly to the refinements where bulk effects were not taken into account, not all O—H bonds were improved upon the introduction of IChE-HAR. Consequently, the MADs of the five O—H bonds were employed to assess the average performance. The MADs of the O—H bond lengths obtained from the refinements performed with and without the embedding scheme are shown at the bottom of Fig. 7 ▸ (blue and red bars, respectively). As can be seen, when IChE-HAR is used, the MAD is reduced by almost half compared with the case in which it is not applied. No significant differences were observed in the *R*_1_, *wR*_2_ or goodness of fit residuals of any of the ten refinements. Additionally, for the intramolecular O⋯H⋯O hydrogen bond of the maleate anion, the two corresponding O⋯H distances, refined with B3LYP_mol_ and B3LYP_IChE_, differ by less than 1 s.u. Finally, in the case of the ADPs of the water molecules (Fig. S2), although lower MADs are also obtained with IChE-HAR, the improvement is moderated as was observed for carbamazepine.

#### 3.3.2. Refinement times

Unfortunately, the ionic nature of these types of system implies that charge convergence between the MM and QM regions requires more cycles, thereby increasing the refinement time. For instance, four cycles were needed for the charge convergence of Mg-HM, in contrast to the three required for carbamazepine. However, it will still be faster than using a cluster of molecules, for which it was not possible to perform converged calculations on the employed computer due to a lack of memory.

### 3.4. Comparison with other embedding schemes

#### 3.4.1. MADs of O—H bond lengths and ADPs of xylitol

The different refinement strategies employed for this system, along with the MADs of the five O—H bonds (Fig. 8 ▸) with respect to the neutron diffraction values, are shown in Fig. 9 ▸. Also included are the MADs obtained with the B3LYP/cc-pVTZ QM/ELMO refinement method, whose values were taken directly from Wieduwilt *et al.* (2020*a* ▸). The cluster employed for B3LYP_clust_ is shown Fig. 8 ▸. As expected, B3LYP_mol_ gives the worst results because crystal environment effects are neglected. The differences between the MADs of B3LYP_IChE_, B3LYP_PCD_ and B3LYP_clust_ are about 1 s.u. of the corresponding O—H refined bond lengths, and therefore their performance in describing the crystalline environment could be considered statistically equivalent. BLYP_IChE_ performed the worst among the point-charge models (larger MAD), while HF_IChE_ yields the best agreement with the neutron diffraction values. The B3LYP/cc-pVTZ QM/ELMO refinement method and HF_IChE_ yield the lowest MADs.

In the case of the ADPs (Fig. S3), again the most pronounced disagreement with neutron diffraction is with B3LYP_mol_. While the differences in the MADs of the rest of the methods are of the order of 1 s.u. of the individual components of the ADPs, there appears to be a tendency for the B3LYP_clust_ method to yield ADPs closer to those of neutrons.

Comparison with the refinement methods that use solid-state electronic structure approaches to generate the electron density is less straightforward, since the method­ologies, resolution, DF, basis sets and other refinement parameters such as extinction are different in each case. Nevertheless, general observations can be extracted from the comparison (Fig. 10 ▸). First, at least for this system, the differences between MADs for the refined O—H bond lengths are more pronounced than between those that use embedding schemes. For the ADPs, the MADs are closer. The most relevant conclusion for this work is that IChE-HAR can achieve an agreement with the neutron diffraction parameters that is comparable to or even better than refinements based on periodic models.

#### 3.4.2. Refinement times

Lastly, a significant contrast is observed in terms of the computation time required for the wavefunction calculations of the methods where this information is available. B3LYP_IChE_ took 0.6 h, while B3LYP_PCD_ took 6.6 h. B3LYP_clust_ took 1.8 h, although the employed cluster was still not enough to cover the first coordination sphere fully. PAW-HAR took 3.33 h with a higher-capacity processor, 32 GB RAM and ten cores. Clearly, in terms of computing time, B3LYP_IChE_ far exceeds the others.

### 3.5. Further considerations of ChE-HAR

One final remark regarding the application of the IChE-HAR method to the alanine crystal structure (data collected at 23 K) is that it can be sensitive to the completeness of the data in cases where the normal HAR procedure shows the same behaviour. By applying B3LYP_mol_ and B3LYP_IChE_ with the def2-TZVP basis set to alanine with full data and with an *F*/σ(*F*) cutoff of 3 [taking as input the data provided by Chu *et al.* (2026 ▸)], we found at least one non-positive-definite ADP. When using the full dataset and B3LYP_IChE_, neither atom refines to a non-positive-definite ADP. We also noticed that the presence of non-positive-definite ADPs for this system was basis-set dependent, with no clear trend. Thus, these effects should be considered when applying embedded HAR methods, since the sensitivity of the refinement results might not only be attributable to the choice of modelling method. Completeness and data quality are crucial, so possible systematic errors [application of σ(*F*) cutoffs, or the *I*/σ^2^(*F*) ratio for different resolution intervals, parameters of the weighting scheme *etc.* (Henn, 2019 ▸; Landeros-Rivera *et al.*, 2021 ▸)] might have strong effects on the resulting models.

## 4. Discussion

### 4.1. ChE-HAR general performance

From the results above it is clear that ignoring the presence of neighbouring molecules causes a decrease in the refined polar *X*—H bond lengths (Figs. 2 ▸, 9 ▸ and S1), in contrast to the procedures in which the crystal environment is taken into account, as expected. The fact that, for instance, the first coordination sphere of carbamazepine could not be completed with the computer equipment used in this work (representative of everyday use) highlights the importance of having an alternative cheaper yet efficient method for modelling the crystalline environment. Since the results obtained for carbamazepine with B3LYP_IChE_ and B3LYP_MChE_ are statistically identical to those of B3LYP_dim_ and B3LYP_clust_, it is shown that the ChE-HAR procedures can be as efficient in refining *X*—H polar bonds as full QM models of the crystalline environment. This is corroborated by the rest of the hydrogen-bonded systems analysed. The comparison between B3LYP_IChE_ and B3LYP_MChE_ for carbamazpine seems to indicate that the use of the UFF Lennard–Jones potential in MChE-HAR does not add any benefit. It is compelling to analyse the effect of utilizing different MM force fields in HAR, which will be studied in future work. However, to avoid introducing more variables, IChE-HAR was chosen for the rest of the systems studied in this work.

On the other hand, regarding the refinement of the ADPs, the analysis of the similarity index (Figs. 3 ▸ and 5 ▸) and the MADs with respect to the neutron values (Figs. S2 and S3) shows that ChE-HAR does not have the same efficiency in describing thermal motion, although it is still better than not considering the crystalline environment at all.

Moreover, in the case of the salt/coordination complex considered here, since the MADs for the O—H bonds of the water molecules coordinated to the metallic centre are very similar when the maleate anion is placed in any position, IChE-HAR could reduce the need to explore various refinement models for this type of system where defining the QM unit is not trivial. The fact that the strong intramolecular O⋯H⋯O hydrogen bond of the maleate anion, classified as resonance-assisted (Malaspina *et al.*, 2020 ▸), is not affected by introducing the crystal field through IChE-HAR suggests that this method is suitable for analysing intermolecular non-covalent interactions but is irrelevant for intramolecular ones.

Finally, comparison with other refinement methods that also introduce crystal-field effects in HAR is not always straightforward. For instance, for QM/ELMO the refinement was performed using *F* instead of *F*^2^ and a different weighting scheme, and, more importantly, the hybrid B3LYP functional is not defined in the same way in *GAUSSIAN09* (Frisch *et al.*, 2009 ▸) (which was used for the wavefunction calculation in the study by Wieduwilt and co-workers) and in *ORCA*. The difference in time performance of B3LYP_IChE_ and B3LYP_PCD_ can partially be attributed to the approximations employed in *ORCA* to speed up the SCF calculations. Although, as mentioned earlier, the nature of solid-state code prevents a more direct comparison with IChE-HAR, it is noteworthy that the latter yields a smaller deviation from the neutron diffraction values for the O—H bond lengths of xylitol than two solid-state programs. In summary, IChE-HAR demonstrates a favourable cost/benefit ratio, as its results are comparable to those of other methods for modelling the crystalline environment at a relatively low computational cost.

### 4.2. Is Hartree–Fock better?

It has been reported that Hartree–Fock (HF) seems to provide better agreement with neutron diffraction values than DFT methods or post-Hartree-Fock methods in systems where strong hydrogen bonds are present, such as in urea, xylitol, carbamazepine or some biomolecules (Wieduwilt *et al.*, 2020*b* ▸; Landeros-Rivera *et al.*, 2023 ▸; Chodkiewicz *et al.*, 2024 ▸; Brüx *et al.*, 2026 ▸), or even a group of 14 amino acids (Brüx *et al.*, 2026 ▸). The authors of these reports have correctly pointed out that this phenomenon may be related to the partitioning scheme and a possible error cancellation, but they have not explored the problem further. We believe that these hypotheses point in the right direction and we propose a more in-depth explanation based on what is known about quantum chemistry. This is important not only for the future development of the ChE-HAR technique but for quantum chemical method development in general.

It is well known that Hirshfeld charges are generally lower than expected (especially for hydrogen atoms) (Davidson & Chakravorty, 1992 ▸; Ayers, 2000 ▸; Bultinck *et al.*, 2007 ▸; Salvador & Ramos-Cordoba, 2013 ▸). Sometimes they have unrealistic values that are not consistent with other partitioning approaches (Saha *et al.*, 2009 ▸) and fall short of reproducing molecular dipole moments (De Proft *et al.*, 2002 ▸). Moreover, Hirshfeld charges are only defined for neutral entities, and thus their application to ionic crystals is questionable (Bultinck *et al.*, 2007 ▸). This has led to the development of methods such as iterative Hirshfeld partitioning and other approaches that correct some of the shortcomings of the original scheme. The reason behind this phenomenon is that Hirshfeld partitioning tends to make atoms appear more neutral and spherical (Ayers, 2000 ▸). It was demonstrated that, as a consequence of weighting factors in Hirshfeld partitioning being positive everywhere, small atoms (like hydrogen) tend to ‘steal’ electrons from their neighbours when they are embedded in electron-rich surroundings (Saha *et al.*, 2009 ▸), explaining why they sometimes show spurious negative charges when the contrary is expected. Therefore, with the Hirshfeld partition more electron density is assigned to hydrogen atoms than should be, especially when it is involved in highly polarized bonds. This could explain why, for these types of system, HAR always underestimates the *X*—H bond lengths, even with correlated methods or periodic conditions. On the other hand, it is established that, due to the lack of static and dynamic correlation, Hartree–Fock overestimates the ionic character of a bond, since the electrons move less far away than they should, and as chemical bonds stretch, the monodeterminantal character of this method also causes the ionic character to predominate. Thus, HF causes hydrogen atoms artificially to ‘donate’ more electron density than they should in polar bonds. Therefore, it appears that the additional electron density assigned by the Hirshfeld partition to hydrogen atoms is offset by the electron density deflected by the Hartree–Fock method, thus allowing a better description of the polarization of the H atoms. When correlated methods are employed, this charge deflection is no longer present and the hydrogen atoms look more spherical. Nevertheless, since these charge transfers are less significant for non-hydrogen atoms (Landeros-Rivera *et al.*, 2023 ▸), whose contribution predominates in the overall electron density, correlated methods will always achieve a better description of the system and therefore lower *R*_1_ values too.

This error cancellation will not always work. For example, for the less polar N—H bond in urea, a better agreement with the neutron diffraction value was reached with pure DFs than with hybrid DFs or with HF (Landeros-Rivera *et al.*, 2023 ▸). In this work, one of the refined C—H bonds of biphenyl obtained with HF_IChE_ reached a value of 1.103 Å, which is 0.020 Å larger than the recommended value of 1.083 Å (Allen & Bruno, 2010 ▸). The IAM (with No-Afix) reached a value of 1.002 Å. Thus, one cannot rely on the error cancellation to occur in all cases to the same extent.

## 5. Conclusion

An electrostatic QMMM-type embedding scheme (ChE-HAR), based on CHELPG charges, has been implemented in *NoSpherA2* to simulate a crystalline environment in Hirshfeld atom refinement. The main advantages of ChE-HAR are its good cost/benefit ratio, automatic operation and database independence. The analysis of the present case studies demonstrates that ChE-HAR has the potential to become a powerful method for modelling strong intermolecular interactions in crystallographic refinements without performing periodic boundary-condition calculations or explicit cluster models, which can become time consuming very quickly. It has been shown that, for refined *X*—H bonds, ChE-HAR can reach the same or even better agreement with neutron diffraction values than the other pure embedding methods, but in significantly shorter times and without the need for external databases. This allows for a more accurate and cheaper determination of refinement parameters, particularly those of hydrogen atoms, which are highly sensitive to these influences.

## Supplementary Material

Supporting information file. DOI: 10.1107/S160057672600717X/ui5039sup1.pdf

## Figures and Tables

**Figure 1:**
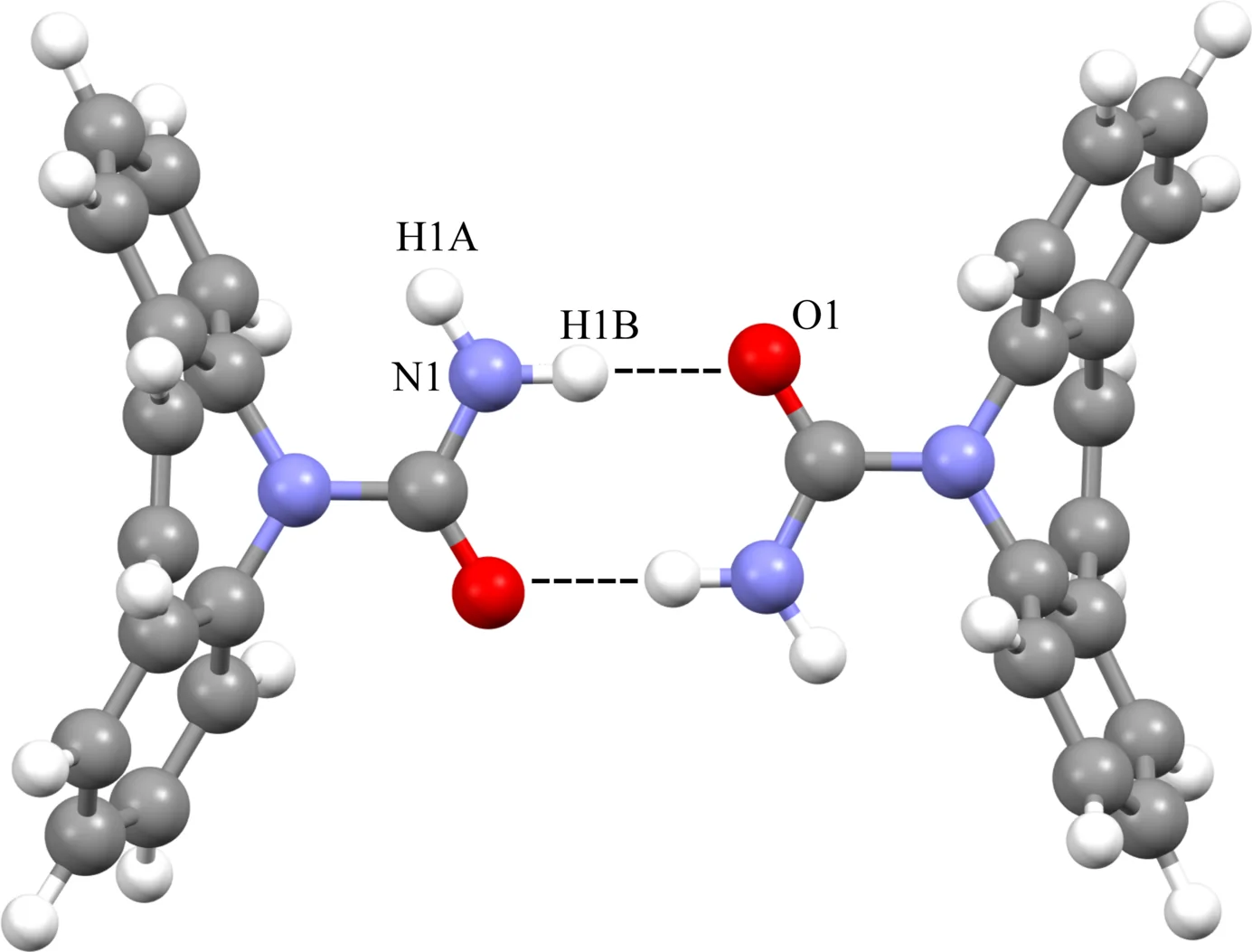
The carbamazepine dimer as found in the crystalline structure. Some relevant atoms are labelled. The N—H⋯O hydrogen bond is shown as a dashed line. The two molecules are symmetry equivalent.

**Figure 2:**
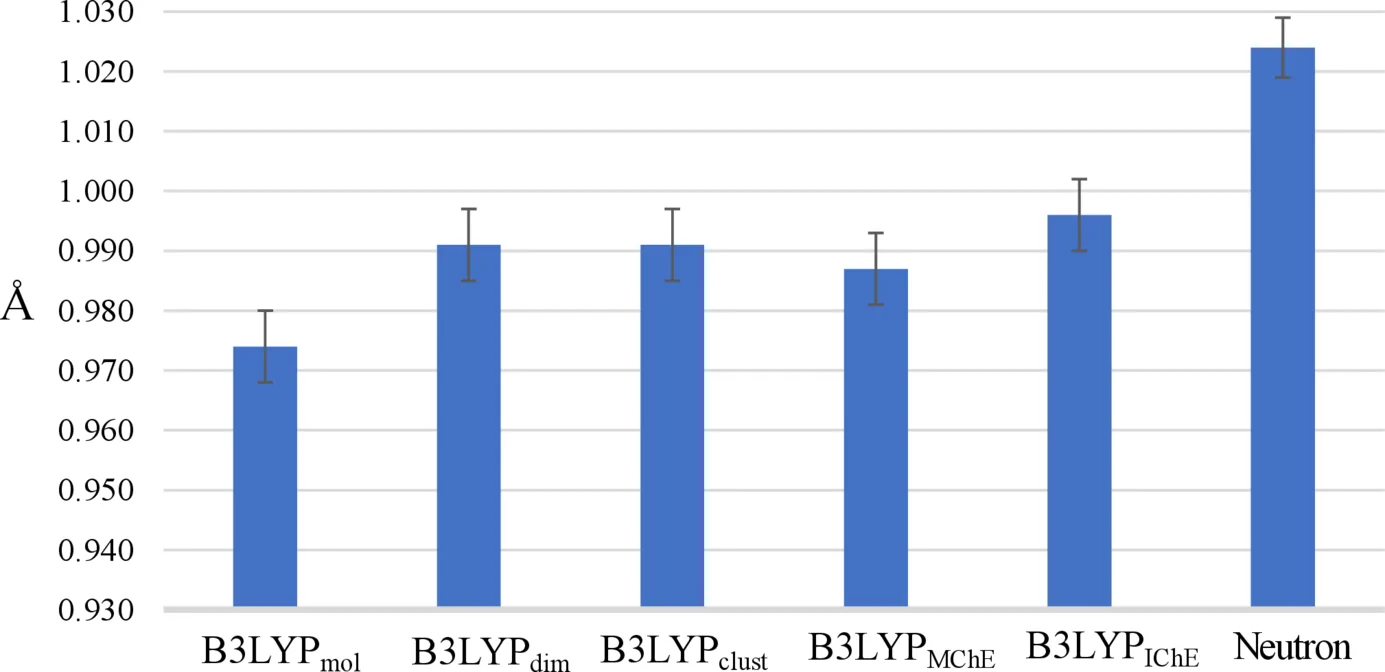
N1—H1*B* refined distances for different HAR procedures. The neutron diffraction value is also shown as a reference. Error bars correspond to standard uncertainties.

**Figure 3:**
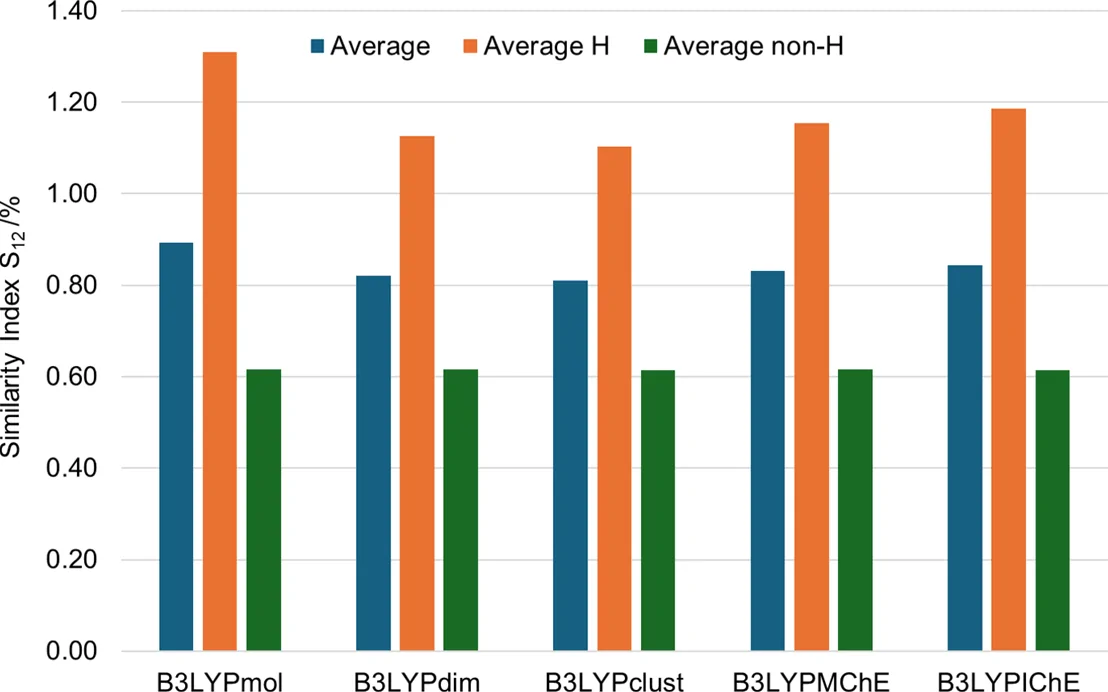
Average values of the ADP similarity index for different HAR refinements compared with the neutron reference structure. The average across all atoms is shown in blue, hydrogen atoms in orange and non-hydrogen atoms in green.

**Figure 4:**
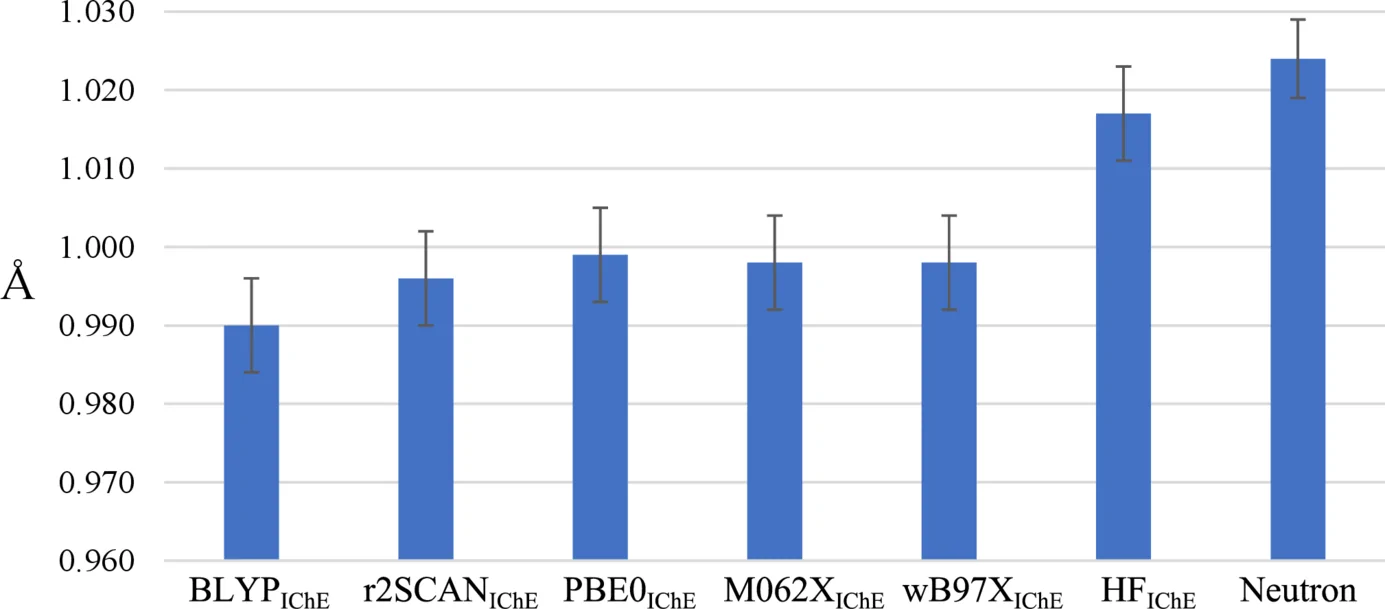
N1—H1*B* refined distances for different DF methods. The neutron diffraction value is also shown as a reference. The relative error with respect to the neutron diffraction value is depicted above each bar. The IChE-HAR procedure was employed in all refinements.

**Figure 5:**
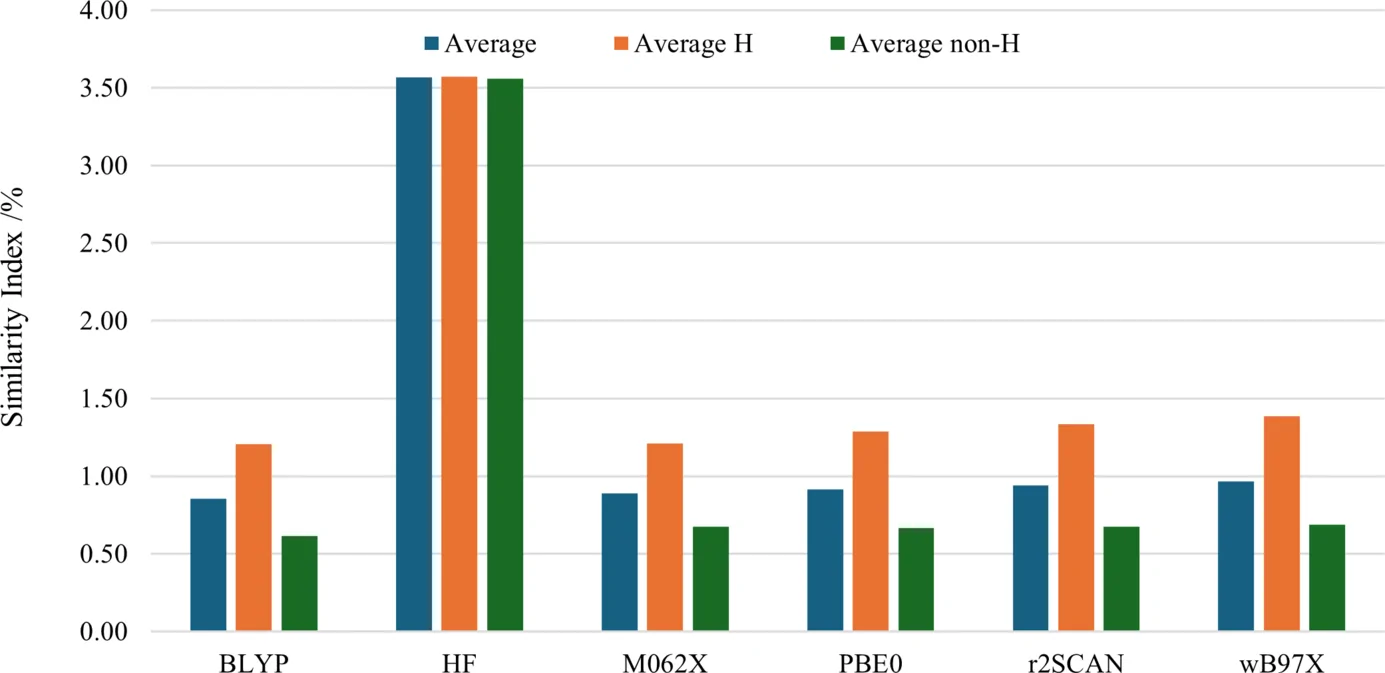
Average values of the ADP similarity index for IChE-HAR refinements using different methods compared with the neutron reference structure. The average across all atoms is shown in blue, hydrogen atoms in orange and non-hydrogen atoms in green.

**Figure 6:**
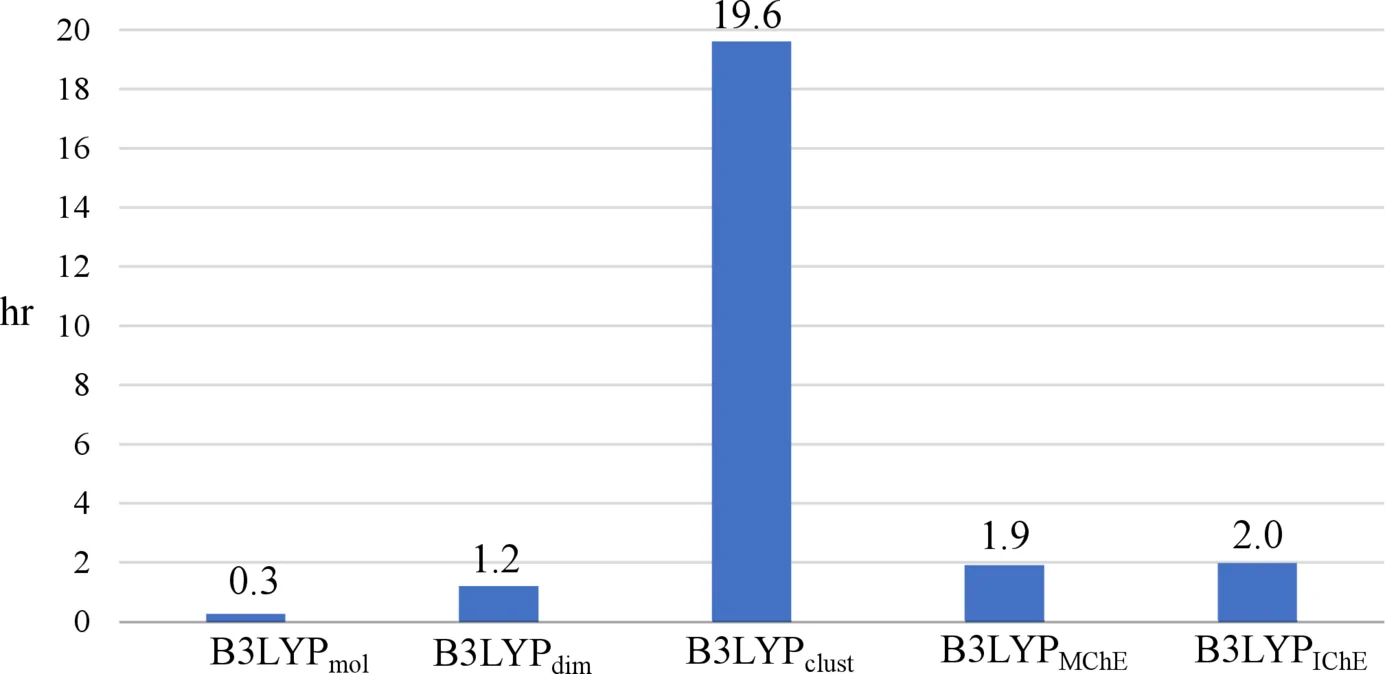
Time (in hours) required for the different HAR procedures for carbamazepine computed with six cores.

**Figure 7:**
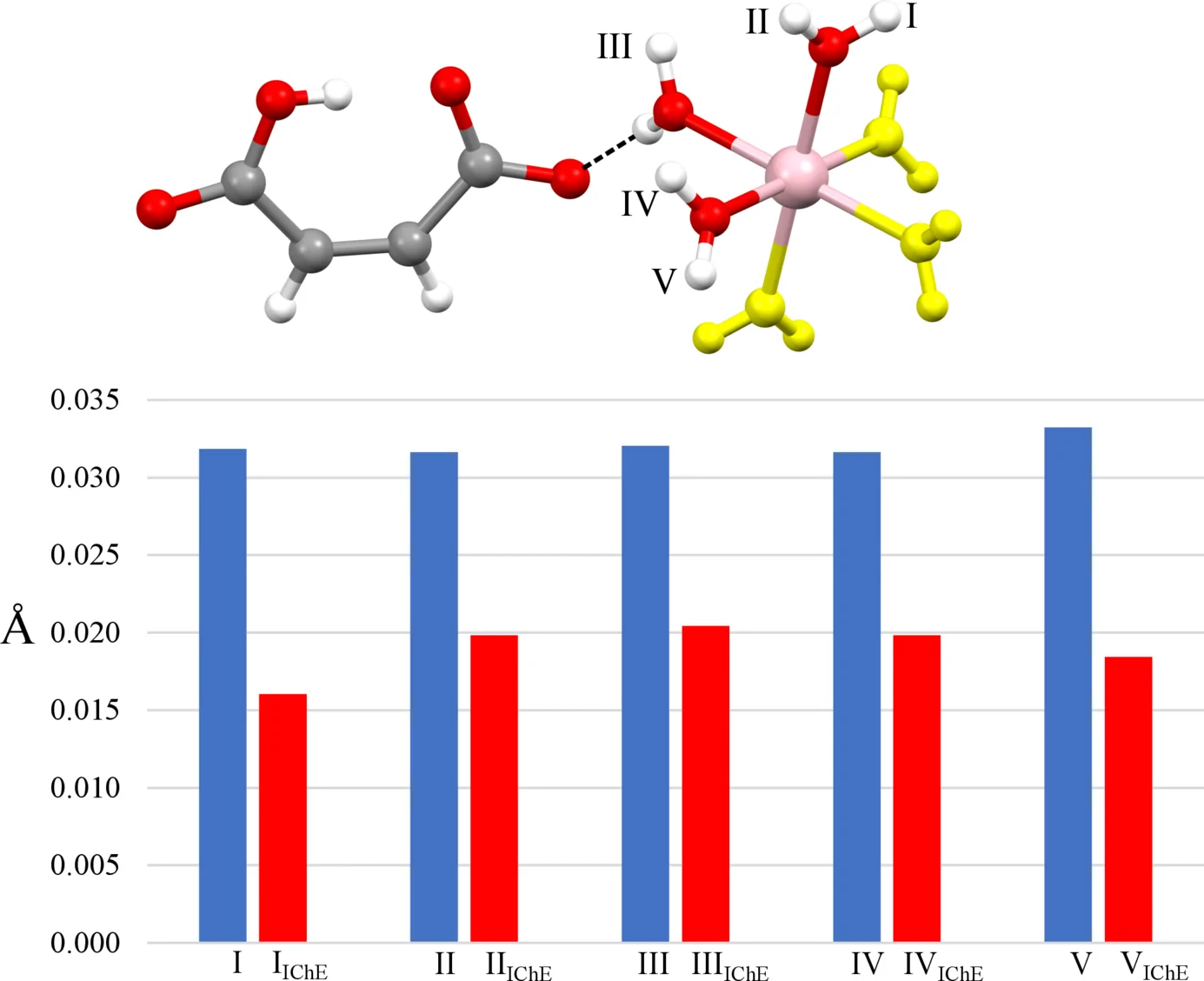
(Top) The structure of the asymmetric unit of Mg-HM (yellow coloured water molecules were symmetry generated). The hydrogen bond formed in the asymmetric unit is shown as a black dashed line. The five positions where the second maleate anion can be placed are indicated by bold Roman numbers **I** to **V**. (Bottom) MADs of the five O—H bond lengths obtained from B3LYP_mol_ (blue bars) and B3LYP_IChE_ (red bars) when the second maleate anion is placed in each different position **I** to **V**.

**Figure 8:**
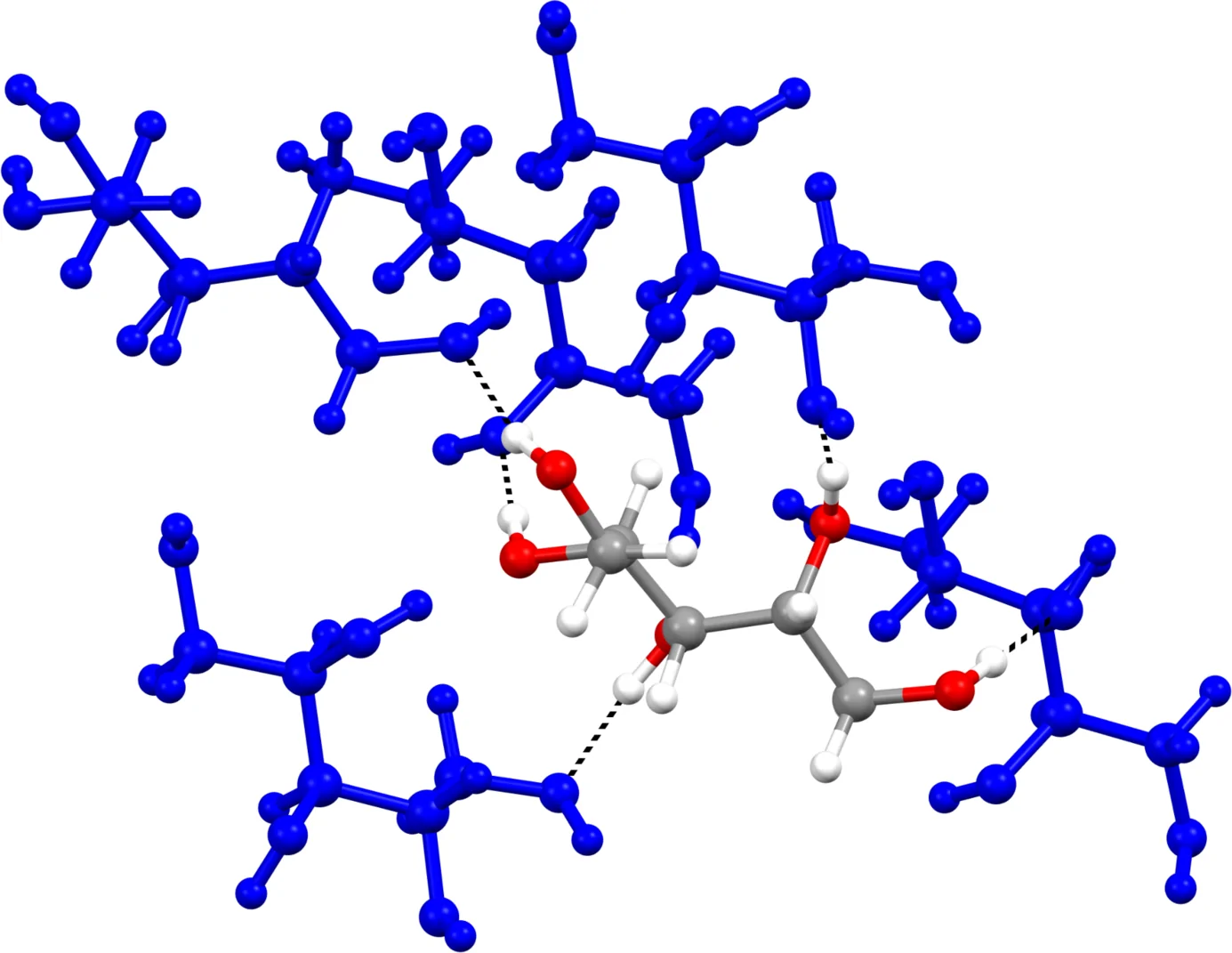
The xylitol cluster employed in the refinements. The blue molecules were symmetry generated. Black dashed lines indicate the five hydrogen bonds.

**Figure 9:**
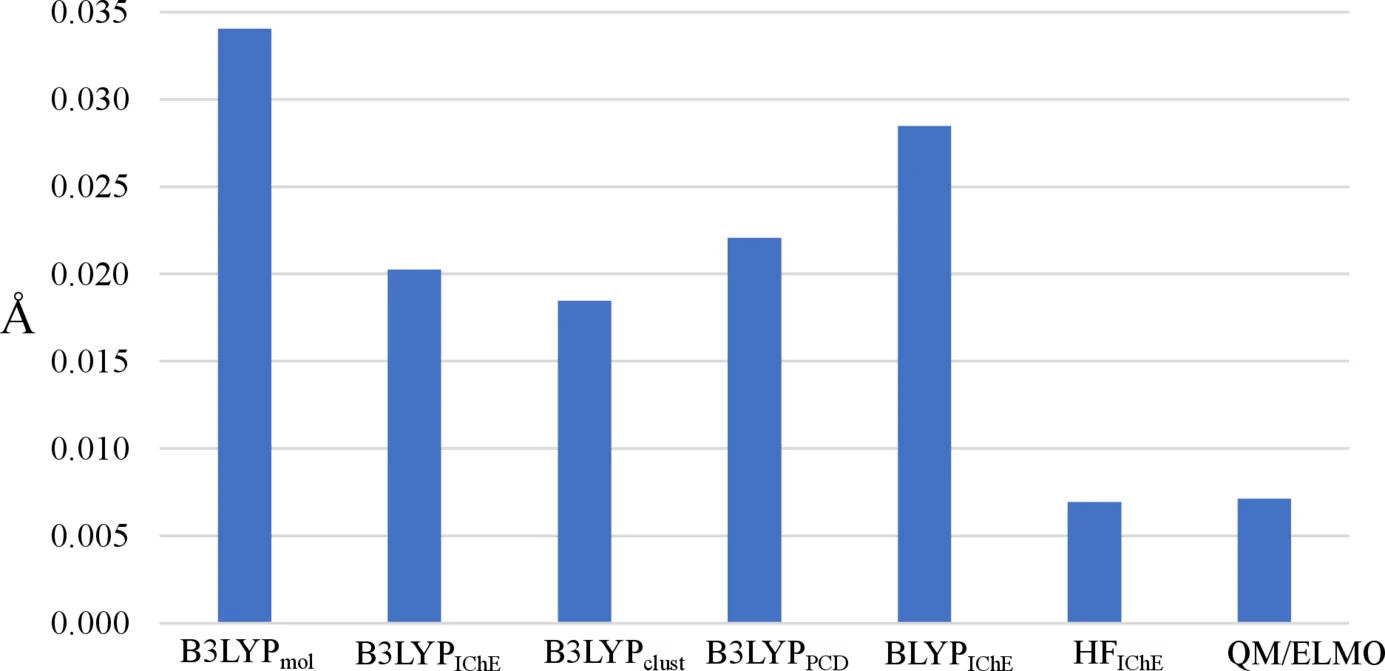
MADs of the five O—H bond lengths of xylitol with respect to neutron diffraction values using different refinement procedures. The MAD computed using the results of Wieduwilt *et al.* (2020*a* ▸), employing the HAR-ELMO methodology, is shown for comparison purposes.

**Figure 10:**
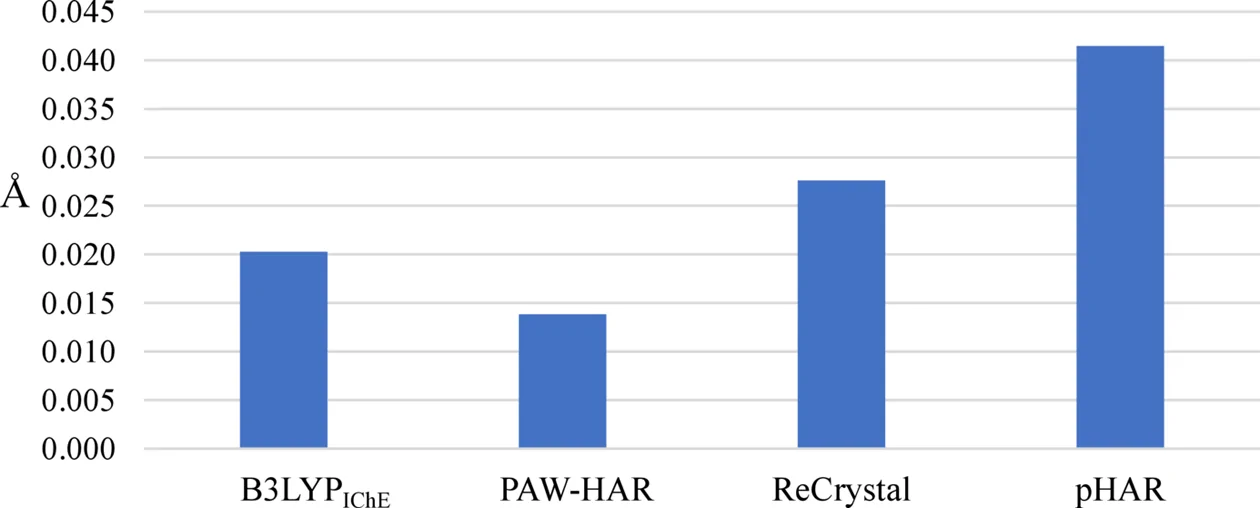
Comparison of the MADs of the five O—H bond lengths of xylitol with respect to neutron diffraction values using B3LYP_IChE_, PAW-HAR, *ReCrystal* and pHAR.
