# Supplementary material for: Application of electrostatic and multi-layer embedding in Hirshfeld atom refinements: a cost-effective approach for approximating bulk effects in crystalline environments
Source: J Appl Crystallogr. 2026 Sep 4;59(Pt 5):1467–77. doi: 10.1107/S160057672600717X (PMC13636035; doi:10.1107/S160057672600717X)
Supplement: Supplementary file 1 [file j-59-01467-sup1.pdf]

JOURNAL OF
APPLIED
CRYSTALLOGRAPHY

Volume 59 (2026)

Supporting information for article:

Application of electrostatic and multi-layer embedding in Hirshfeld atom refinements: a cost-effective approach for approximating bulk effects in crystalline environments

Bruno Landeros-Rivera and Florian Kleemiss

Supplementary information for "Application of Electrostatic QM/MM-type And Multi-layer Embedding in Hirshfeld Atom Refinement Inside NoSpherA2

Bruno Landeros-Rivera[a] and Florian Kleemiss [b*]

[a]Departamento de Química Inorgánica, Facultad de Química, Universidad Nacional Autónoma de México, Mexico, and [b]RWTH Aachen University, Institute of Inorganic Chemistry, Landoltweg 1a, 52074 Aachen, Germany.

E-mail: florian.kleemiss@ac.rwth-aachen.de

Table S1. Refined bond X-H bond lengths of carbamazepine with the B3LYP/def2-TZVP level of theory for different HAR strategies. Errors with respect to neutron diffraction are shown in red (defined as the neutron minus the X-ray refined bond distances).

Atom1	Atom2	Mol	Dev.	Dim	Dev.	Clust	Dev.	IChE	Dev.	MChE	Dev.	Neutron
C11	H11	1.094	-0.005	1.094	-0.005	1.091	-0.002	1.105	-0.016	1.092	-0.003	1.089
C12	H12	1.075	0.017	1.075	0.017	1.079	0.013	1.080	0.012	1.080	0.012	1.092
C13	H13	1.078	0.011	1.078	0.011	1.086	0.003	1.087	0.002	1.083	0.006	1.089
C14	H14	1.079	0.009	1.080	0.008	1.078	0.010	1.068	0.020	1.078	0.010	1.088
N1	H1A	0.977	0.022	0.974	0.025	0.978	0.021	0.977	0.022	0.979	0.020	0.999
C3	H3	1.071	0.018	1.070	0.019	1.070	0.019	1.060	0.029	1.070	0.019	1.089
C4	H4	1.081	0.005	1.081	0.005	1.082	0.004	1.083	0.003	1.082	0.004	1.086
C5	H5	1.083	0.009	1.082	0.010	1.083	0.009	1.100	-0.008	1.083	0.009	1.092
C6	H6	1.078	0.011	1.078	0.011	1.080	0.009	1.107	-0.018	1.082	0.007	1.089
C8	H8	1.083	0.011	1.082	0.012	1.082	0.012	1.082	0.012	1.081	0.013	1.094
C9	H9	1.074	0.014	1.074	0.014	1.084	0.004	1.097	-0.009	1.083	0.005	1.088

Table S2. Refined bond X-H bond lengths of carbamazepine with different density functionals. Errors with respect to neutron diffraction are shown in red (defined as the neutron minus the X-ray refined bond distances).

Atom1	Atom2	BLYP	Dev.	r2SCAN	Dev.	PBE0	Dev.	M062X	Dev.	wB97X	Dev.	HF	Dev.
C11	H11	1.102	-0.013	1.106	-0.017	1.107	-0.018	1.106	-0.017	1.106	-0.017	1.110	-0.021
C12	H12	1.076	0.016	1.080	0.012	1.082	0.010	1.080	0.012	1.081	0.011	1.089	0.003
C13	H13	1.083	0.006	1.088	0.001	1.090	-0.001	1.088	0.001	1.090	-0.001	1.099	-0.010
C14	H14	1.064	0.024	1.068	0.020	1.070	0.018	1.068	0.020	1.070	0.018	1.076	0.012
N1	H1A	0.973	0.026	0.979	0.020	0.981	0.018	0.980	0.019	0.980	0.019	0.991	0.008
C3	H3	1.058	0.031	1.061	0.028	1.063	0.026	1.060	0.029	1.062	0.027	1.063	0.026
C4	H4	1.080	0.006	1.083	0.003	1.085	0.001	1.083	0.003	1.085	0.001	1.088	-0.002
C5	H5	1.098	-0.006	1.100	-0.008	1.102	-0.010	1.101	-0.009	1.101	-0.009	1.104	-0.012
C6	H6	1.104	-0.015	1.107	-0.018	1.109	-0.020	1.107	-0.018	1.108	-0.019	1.112	-0.023
C8	H8	1.080	0.014	1.082	0.012	1.084	0.010	1.082	0.012	1.083	0.011	1.083	0.011
C9	H9	1.093	-0.005	1.097	-0.009	1.099	-0.011	1.098	-0.010	1.099	-0.011	1.106	-0.018
**MAE**		**0.0147**		**0.0135**		**0.0130**		**0.0136**		**0.0131**		**0.0133**	

Table S3. Absolute errors (Å) of the O-H bonds of *Mg-HM*, with respect to neutron diffraction values, for various refinements. The corresponding hydrogen position where the second maleate anion was placed is indicated in parentheses.

Bond	Refinement **I**	Refinement **II**	Refinement **III**	Refinement **IV**	Refinement **V**
O1-H2	0.0006	0.0014	0.0026	0.0086	0.0046
O2-H2	0.0000	0.0020	0.0020	0.0090	0.0040
O5-H5a (I)	0.0197	0.0357	0.0337	0.0127	0.0297
O5-H5b (II)	0.0384	0.0264	0.0364	0.0384	0.0134
O6-H6a (III)	0.0323	0.0323	0.0203	0.0073	0.0053
O6-H6b	0.0045	0.0035	0.0075	0.0095	0.0135
O7-H7a (VI)	0.0400	0.0330	0.0420	0.0390	0.0170
O7-H7b (V)	0.0288	0.0308	0.0278	0.0018	0.0268

Bond	Ref. $\mathbf{I}_{IChE}$	Ref. $\mathbf{II}_{ChE}$	Ref. $\mathbf{III}_{ChE}$	Ref. $\mathbf{IV}_{ChE}$	Ref. $\mathbf{V}_{ChE}$
O1-H2	0.0036	0.0086	0.0046	0.0014	0.0014
O2-H2	0.0030	0.0090	0.0050	0.0020	0.0020
O5-H5a (I)	0.0437	0.0127	0.0247	0.0357	0.0357
O5-H5b (II)	0.0064	0.0384	0.0194	0.0264	0.0344
O6-H6a (III)	0.0023	0.0073	0.0283	0.0323	0.0343
O6-H6b	0.0095	0.0095	0.0065	0.0035	0.0035
O7-H7a (VI)	0.0160	0.0390	0.0190	0.0330	0.0450
O7-H7b (V)	0.0118	0.0018	0.0108	0.0308	0.0168

Table S4. Errors (defined as the neutron minus the X-ray refined bond distances) of the O-H bond lengths of Xylitol (Å).

Bond	$B3LYP_{mol}$	$B3LYP_{IChE}$	$B3LYP_{clust}$	$B3LYP_{PCD}$	$BLYP_{IChE}$	HF_{IChE}	QM/ELMO
O-H1	0.0400	0.0230	0.0300	0.0270	0.0330	-0.0090	0.0020
O-H2	0.0340	0.0190	0.0220	0.0240	0.0270	-0.0040	0.0070
O-H3	0.0253	0.0133	0.0033	0.0143	0.0203	-0.0077	-0.0147
O-H4	0.0210	0.0120	0.0080	0.0110	0.0190	-0.0100	-0.0010
O-H5	0.0500	0.0340	0.0290	0.0340	0.0430	0.0040	0.0110

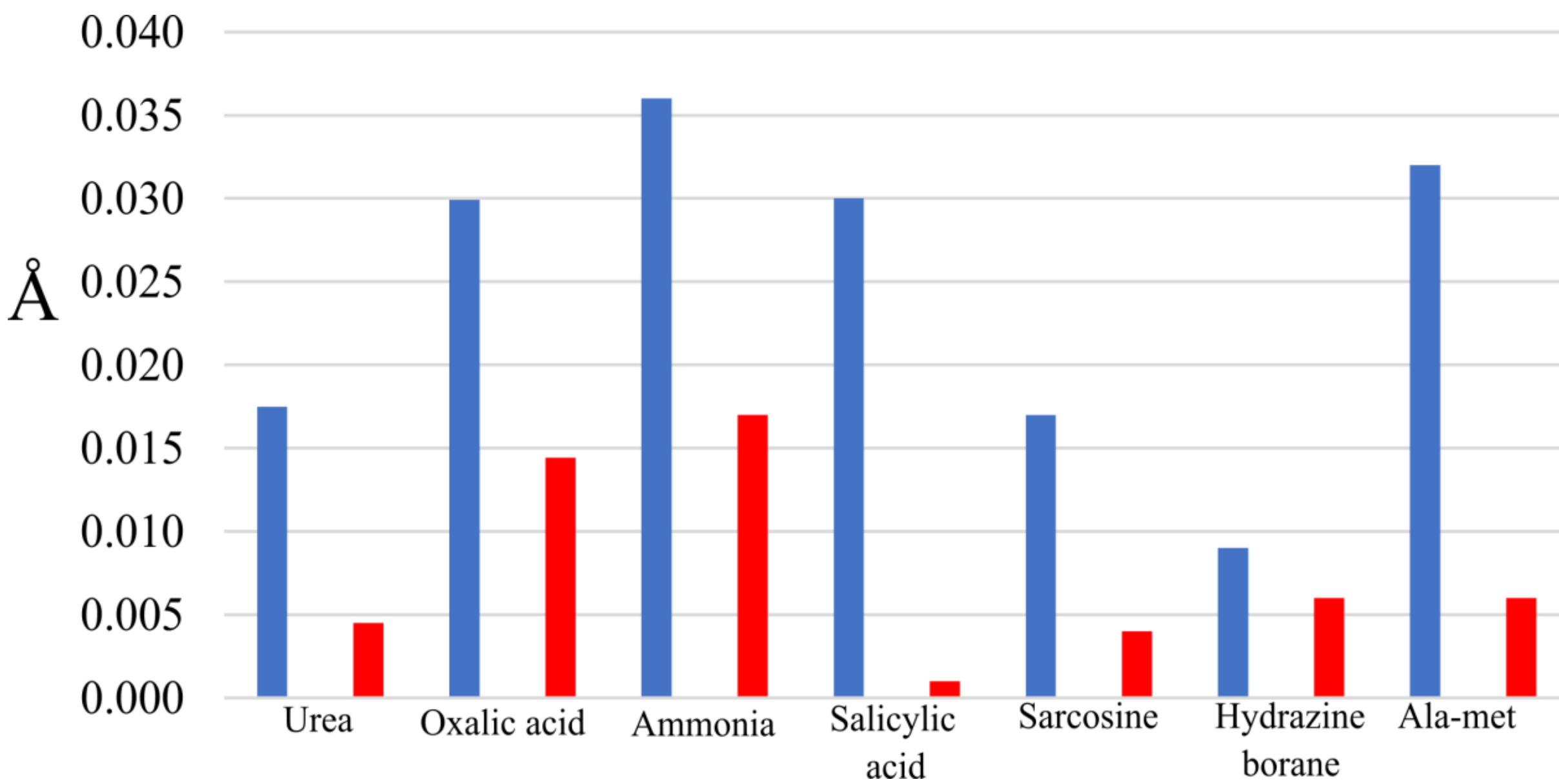

Figure S1. MADs of the hydrogen-bonded systems with respect to neutron diffraction references for $B3LYP_{mol}$ (blue bars) and $B3LYP_{IChE}$ (red bars).

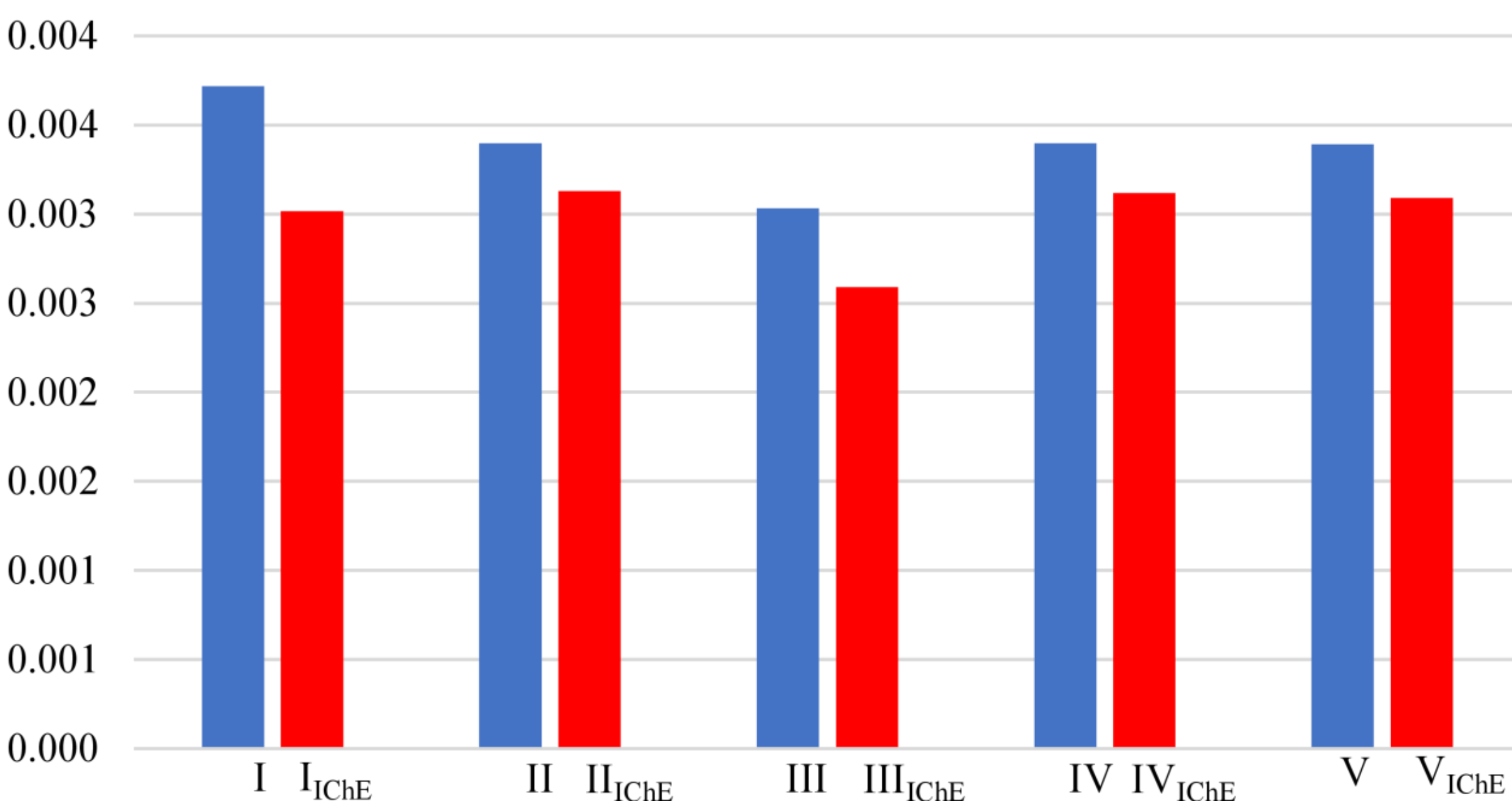

Figure S2. MADs of ADPS of the five H atoms (indicated in Figure 5) obtained from the refinements with (red bars) and without (blue bars) employing the embedding scheme, when the second maleate anion is placed in each different position.

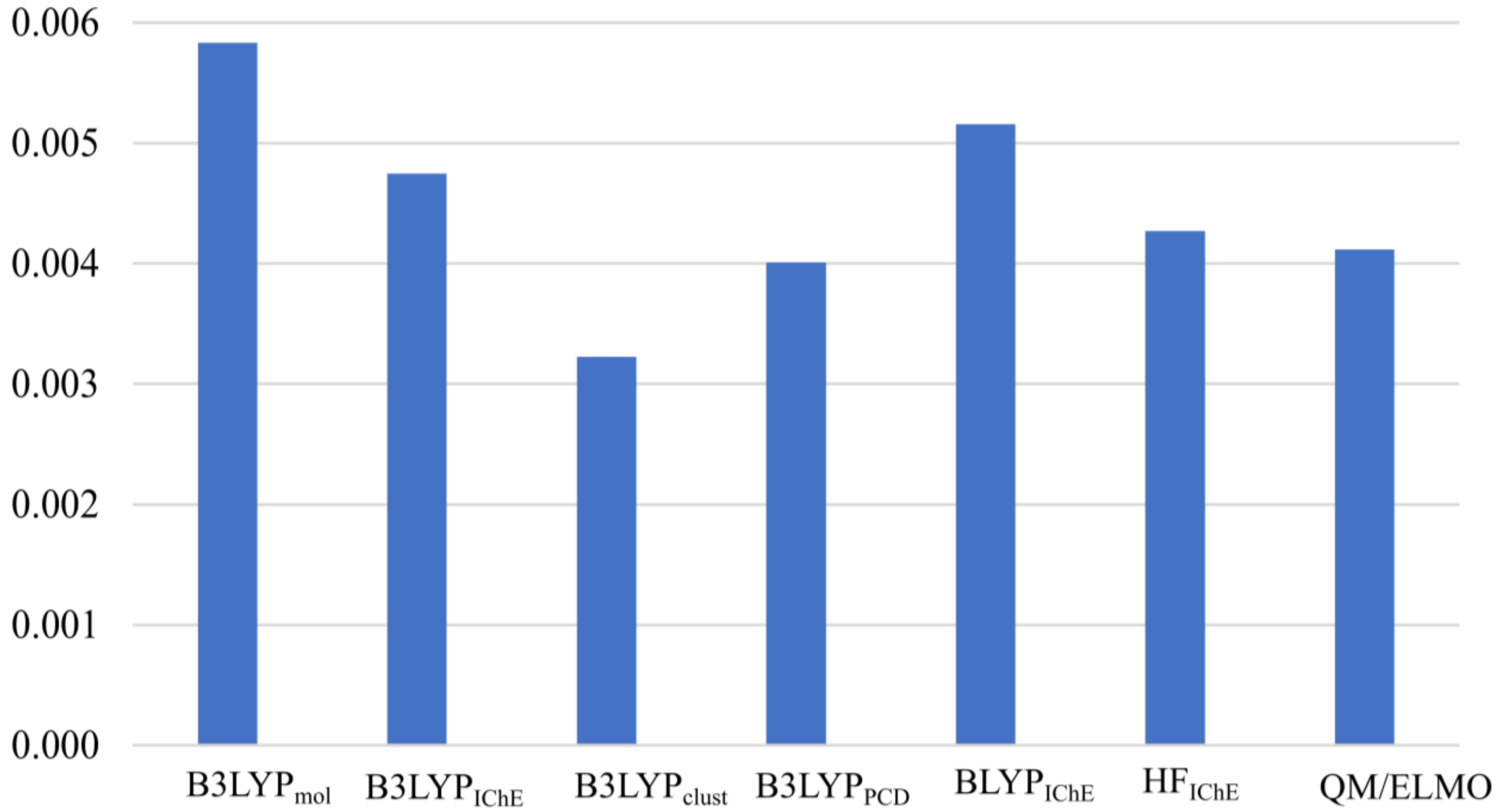

Figure S3. MAD of the ADPs (Å^2) of the five H atoms of xylitol (Figure 7) using different refinement procedures. The MAD computed with the results of Wieduwilt *et al.* (Wieduwilt et al., 2020), employing the HAR-ELMO methodology, is shown for comparison purposes.

Table S5. Similarity Index (S12) in % for all atoms in carbamazepine using different HAR models against the Neutron reference values.

Atoms	mol	dim	clust	MChE	IChE
**O1**	1.35	1.37	1.38	1.38	1.37
**N1**	0.61	0.59	0.59	0.59	0.59
**N2**	0.88	0.88	0.87	0.87	0.86
**C1**	0.73	0.74	0.73	0.73	0.73
**C2**	0.73	0.73	0.73	0.73	0.73
**C3**	0.43	0.43	0.43	0.43	0.43
**C4**	0.43	0.43	0.43	0.43	0.43
**C5**	0.38	0.38	0.38	0.38	0.37
**C6**	0.27	0.27	0.27	0.27	0.29
**C7**	0.69	0.69	0.69	0.69	0.69
**C8**	0.59	0.59	0.59	0.59	0.60
**C9**	0.77	0.77	0.76	0.77	0.76
**C10**	0.82	0.81	0.81	0.81	0.81
**C11**	0.42	0.42	0.42	0.42	0.41
**C12**	0.64	0.64	0.64	0.64	0.64
**C13**	0.33	0.33	0.33	0.33	0.33
**C14**	0.22	0.22	0.22	0.22	0.22
**C15**	0.79	0.79	0.79	0.80	0.80
**H1A**	3.40	3.69	3.36	3.22	3.56
**H1B**	3.61	0.99	0.99	1.99	1.54
**H3**	0.97	0.75	0.75	0.75	0.72
**H4**	0.15	0.21	0.23	0.19	0.25
**H5**	0.21	0.36	0.37	0.37	0.83
**H6**	3.81	3.89	3.98	3.65	3.69
**H8**	0.77	0.77	0.91	0.91	0.86
**H9**	0.61	0.58	0.44	0.45	0.63
**H11**	0.91	0.96	1.10	1.15	0.84
**H12**	0.44	0.44	0.35	0.34	0.28
**H13**	0.40	0.44	0.32	0.39	0.34
**H14**	0.43	0.44	0.44	0.44	0.69
**Average**	**0.89**	**0.82**	**0.81**	**0.83**	**0.84**
**Average H**	**1.31**	**1.13**	**1.10**	**1.15**	**1.19**
**Average non-H**	**0.62**	**0.62**	**0.61**	**0.62**	**0.61**

Table S6. Similarity Index (S12) in % for all atoms using different embedding models against the monomer calculation values.

Atoms	dim	clust	MChE	IChE
**O1**	0.00	0.00	0.00	0.00
**N1**	0.00	0.00	0.00	0.00
**N2**	0.00	0.00	0.00	0.00
**C1**	0.00	0.00	0.00	0.00
**C2**	0.00	0.00	0.00	0.00
**C3**	0.00	0.00	0.00	0.00
**C4**	0.00	0.00	0.00	0.00
**C5**	0.00	0.00	0.00	0.00
**C6**	0.00	0.00	0.00	0.00
**C7**	0.00	0.00	0.00	0.00
**C8**	0.00	0.00	0.00	0.00
**C9**	0.00	0.00	0.00	0.00
**C10**	0.00	0.00	0.00	0.00
**C11**	0.00	0.00	0.00	0.00
**C12**	0.00	0.00	0.00	0.00
**C13**	0.00	0.00	0.00	0.00
**C14**	0.00	0.00	0.00	0.00
**C15**	0.00	0.00	0.00	0.00
**H1A**	0.02	0.02	0.01	0.05
**H1B**	1.06	1.06	0.37	1.01
**H3**	0.04	0.04	0.04	0.16
**H4**	0.02	0.02	0.01	0.06
**H5**	0.03	0.04	0.06	0.56
**H6**	0.02	0.04	0.04	0.99
**H8**	0.03	0.04	0.03	0.61
**H9**	0.00	0.04	0.04	0.22
**H11**	0.00	0.06	0.06	0.10
**H12**	0.02	0.11	0.06	0.31
**H13**	0.00	0.01	0.01	0.21
**H14**	0.00	0.02	0.02	0.02
**Average**	**0.04**	**0.05**	**0.03**	**0.14**
**Average H**	**0.10**	**0.13**	**0.06**	**0.36**
**Average non-H**	**0.00**	**0.00**	**0.00**	**0.00**

Table S7. Similarity Index (S12) in % for all atoms using different embedding models against the monomer calculation values.

Atoms	B3LYP	BLYP	HF	M062X	PBE0	r2SCAN	wB97X	PBE
**O1**	1.37	1.35	1.44	1.39	1.40	1.41	1.41	1.41
**N1**	0.59	0.58	0.67	0.62	0.62	0.62	0.62	0.58
**N2**	0.86	0.83	0.98	0.90	0.90	0.90	0.90	0.97
**C1**	0.73	0.71	0.84	0.79	0.78	0.79	0.77	0.77
**C2**	0.73	0.70	0.87	0.79	0.77	0.77	0.77	0.73
**C3**	0.43	0.41	0.50	0.46	0.46	0.45	0.45	0.48
**C4**	0.43	0.42	0.50	0.47	0.46	0.46	0.46	0.47
**C5**	0.37	0.36	0.43	0.40	0.39	0.40	0.39	0.38
**C6**	0.29	0.28	0.34	0.31	0.31	0.31	0.31	0.31
**C7**	0.69	0.67	0.81	0.74	0.73	0.73	0.73	0.71
**C8**	0.60	0.58	0.67	0.63	0.62	0.62	0.62	0.58
**C9**	0.76	0.74	0.85	0.80	0.79	0.79	0.79	0.73
**C10**	0.81	0.78	0.94	0.87	0.85	0.86	0.85	0.87
**C11**	0.41	0.40	0.49	0.45	0.44	0.44	0.44	0.50
**C12**	0.64	0.62	0.73	0.68	0.67	0.67	0.66	0.69
**C13**	0.33	0.32	0.40	0.36	0.35	0.35	0.35	0.33
**C14**	0.22	0.21	0.27	0.24	0.23	0.23	0.23	0.23
**C15**	0.80	0.77	0.92	0.86	0.84	0.85	0.84	0.77
**H1A**	3.56	3.92	4.18	3.57	3.69	3.57	3.69	4.18
**H1B**	1.54	1.73	1.40	1.41	1.54	1.54	1.54	1.60
**H3**	0.72	0.39	1.23	0.82	0.89	0.81	0.92	0.89
**H4**	0.25	0.23	1.04	0.18	0.20	0.20	0.24	0.25
**H5**	0.83	0.83	0.66	1.18	1.18	1.18	0.86	1.22
**H6**	3.69	3.68	4.29	4.25	4.46	4.46	4.53	3.03
**H8**	0.86	0.96	2.47	1.00	1.00	1.00	0.99	0.81
**H9**	0.63	0.73	0.41	0.56	0.57	0.51	0.45	0.70
**H11**	0.84	0.79	1.20	0.98	0.99	1.05	1.05	0.99
**H12**	0.28	0.33	0.79	0.32	0.39	0.44	0.39	0.39
**H13**	0.34	0.31	0.59	0.40	0.32	0.37	0.32	0.35
**H14**	0.69	0.63	1.25	0.60	0.52	0.60	0.60	0.67
**Average**	**0.84**	**0.84**	**1.07**	**0.90**	**0.91**	**0.91**	**0.91**	**0.89**
**Average H**	**1.19**	**1.21**	**1.63**	**1.27**	**1.31**	**1.31**	**1.30**	**1.26**
**Average non-H**	**0.61**	**0.60**	**0.70**	**0.65**	**0.65**	**0.65**	**0.64**	**0.64**

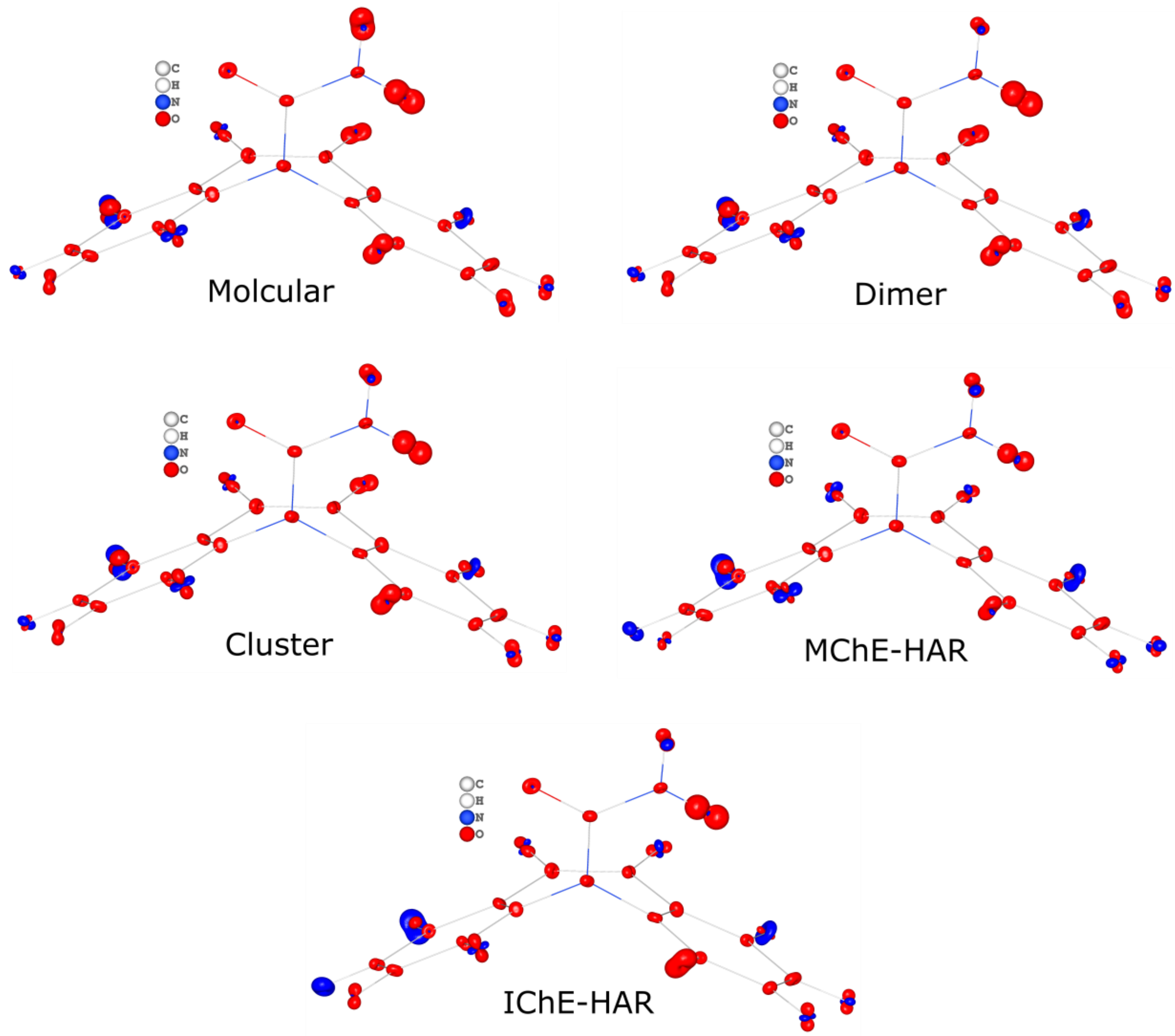

Figure S4. Peanut plots (RMSD) of the differences between carbamazepine refinements using techniques indicated below the molecule against the neutron reference structure scaled by a factor of 5. Positive regions (blue) indicate higher displacement of the nuclear position in the neutron structure, while negative regions (red) indicate higher displacement values for the refined X-ray structure.

Table S8. Similarity Index (S12) in % for all atoms between molecular and embedding models for Alanine Methionine.

**Alanine-Methionine**	**Molecule-IChE**
**N1**	0.01
**H1a**	1.51
**H1b**	0.69
**H1c**	0.29
**C1A**	0.02
**H1Aa**	0.87
**C1B**	0.00
**H1Ba**	0.09
**H1Bb**	0.17
**H1Bc**	0.38
**C1**	0.00
**O1**	0.00
**N2**	0.00
**C2A**	0.01
**H2A**	2.64
**C2B**	0.00
**H2Ba**	0.11
**H2Bb**	0.07
**C2G**	0.00
**H2Ga**	0.19
**H2Gb**	0.55
**S2D**	0.00
**C2E**	0.00
**H2Ea**	0.29
**H2Eb**	0.04
**H2Ec**	0.10
**C2**	0.00
**O2**	0.00
**O22**	0.00
**H2**	0.20
**Average**	**0.27**
**Average H**	**0.51**
**Average non-H**	**0.00**

Table S9. Similarity Index (S12) in % for all atoms between molecular and embedding models for the asymmetric unit of biphenyl.

**Biphenyl**	**Molecule-IChE**
**C(1)**	0.00
**C(2)**	0.00
**H(2)**	0.20
**C(3)**	0.00
**H(3)**	0.01
**C(4)**	0.00
**H(4)**	0.04
**C(5)**	0.00
**H(5)**	0.15
**C(6)**	0.00
**H(6)**	0.08
**Average**	**0.04**
**Average H**	**0.10**
**Average non-H**	**0.00**

Table S11. Similarity Index (S12) in % for all atoms between molecular and embedding models for the asymmetric unit of hydrazine borane.

**Hydrazine borane**	**Molecule-IChE**
**N1**	0.00
**N2**	0.00
**B**	0.00
**H1a**	0.22
**H1b**	0.12
**H2a**	0.04
**H2b**	0.05
**Ha**	0.03
**Hb**	0.03
**Hc**	0.03
**Average**	**0.05**
**Average H**	**0.07**
**Average non-H**	**0.00**

Table S12. Similarity Index (S12) in % for all atoms between molecular and embedding models for the asymmetric unit of ammonia.

**Ammonia**	**Molecule-IChE**
**N**	0.00
**H1**	0.08

Table S13. Similarity Index (S12) in % for all atoms between molecular and embedding models for the asymmetric unit of diborane.

**Hydrazine borane**	**Molecule-IChE**
**B1**	0.00
**H1**	0.00
**H2**	0.00
**H4**	0.00

Table S14. Similarity Index (S12) in % for all atoms between molecular and embedding models for the asymmetric unit of sarcosine.

**Sarcosine**	**Molecule-IChE**
**O(1)**	0.00
**O(2)**	0.00
**N(1)**	0.00
**C(1)**	0.00
**C(2)**	0.01
**C(3)**	0.00
**H(3a)**	0.23
**H(2a)**	2.27
**H(2b)**	1.07
**H(3b)**	0.46
**H(3c)**	0.36
**H(1a)**	0.88
**H(1b)**	1.11
**Average**	**0.49**
**Average H**	**0.91**
**Average non-H**	**0.00**

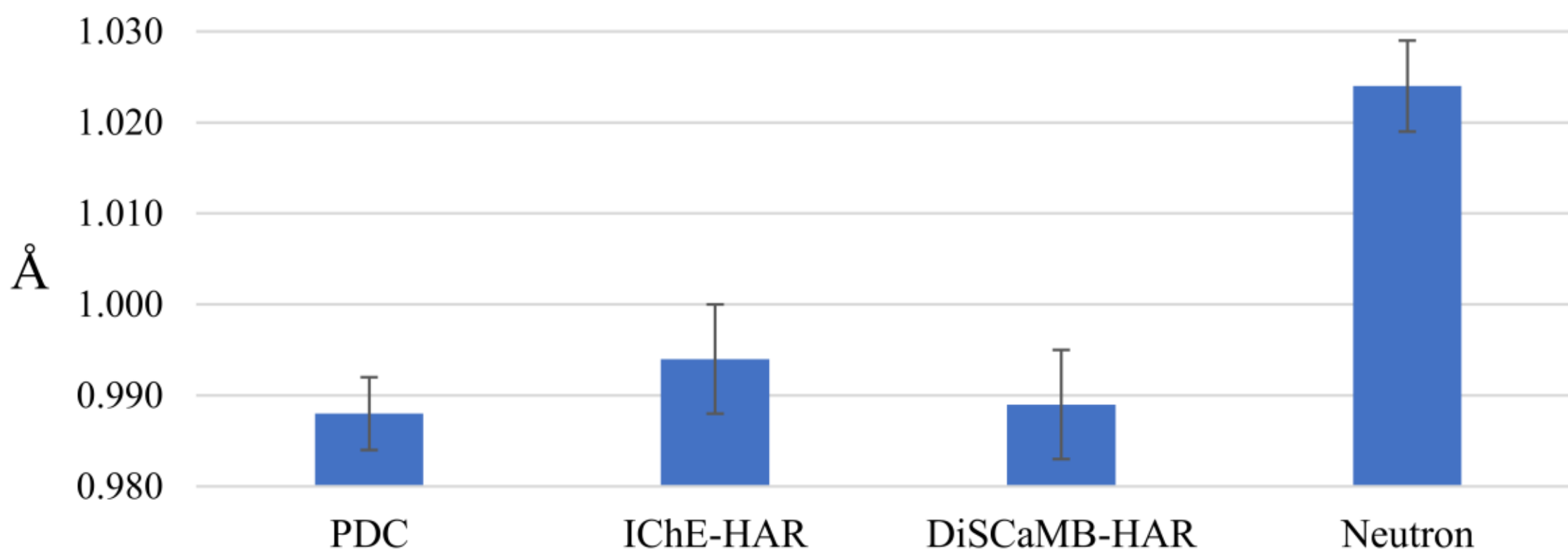

Figure S5. N1-H1B refined distances for different HAR procedures, employing the BLYP/cc-pVTZ level of theory. Neutron diffraction value is also shown as reference. Error bars correspond to standard uncertainties.
